# Bis{[μ-bis­(diphenyl­arsino)methane-1:2κ^2^ *As*:*As*′]nona­carbonyl-1κ^3^ *C*,2κ^3^ *C*,3κ^3^ *C*-[tris­(4-chloro­phen­yl)phosphine-3κ*P*]-*triangulo*-triruthenium(0)} chloro­form monosolvate

**DOI:** 10.1107/S1600536809053884

**Published:** 2009-12-24

**Authors:** Omar bin Shawkataly, Imthyaz Ahmed Khan, Chin Sing Yeap, Hoong-Kun Fun

**Affiliations:** a Chemical Sciences Programme, School of Distance Education, Universiti Sains Malaysia, 11800 USM, Penang, Malaysia; b X-ray Crystallography Unit, School of Physics, Universiti Sains Malaysia, 11800 USM, Penang, Malaysia

## Abstract

The asymmetric unit of the title *triangulo*-triruthenium compound, 2[Ru_3_(C_25_H_22_As_2_)(C_18_H_12_Cl_3_P)(CO)_9_]·CHCl_3_, consists of two mol­ecules (*A* and *B*) of the *triangulo*-triruthenium complex and one mol­ecule of chloro­form solvent. The bis­(diphenyl­arsino)methane ligand bridges an Ru—Ru bond and the monodentate phosphine ligand bonds to the third Ru atom. Both the phosphine and arsine ligands are equatorial with respect to the Ru_3_ triangle. In addition, each Ru atom carries one equatorial and two axial terminal carbonyl ligands. The three phosphine-substituted benzene rings make dihedral angles of 73.5 (3), 57.2 (3) and 75.7 (3)° with each other in mol­ecule *A*, while these angles are 60.7 (3), 86.8 (3) and 54.9 (3)° in mol­ecule *B*. The dihedral angles between the two benzene rings are 87.3 (3) and 89.6 (3)° for the two diphenyl­arsino groups in mol­ecule *A* and 85.6 (3) and 87.7 (3)° in mol­ecule *B*. In the crystal packing, the mol­ecules are linked into a three-dimensional framework *via* inter­molecular C—H⋯O and C—H⋯Cl hydrogen bonds. Weak inter­molecular C—H⋯π inter­actions furture stabilize the crystal structure. The crystal studied was an inversion twin, the refined ratio of twin components being 0.480 (7):0.520 (7).

## Related literature

For general background to *triangulo*-triruthenium derivatives, see: Bruce *et al.* (1985 ▶, 1988*a* ▶,*b* ▶). For related structures, see: Shawkataly *et al.* (1998 ▶, 2004 ▶, 2009 ▶). For the synthesis of μ-bis­(diphenyl­arsino)methane­deca­carbonyl­triruthenium(0), see: Bruce *et al.* (1983 ▶). For stability of the temperature controller used for the data collection, see: Cosier & Glazer (1986 ▶).
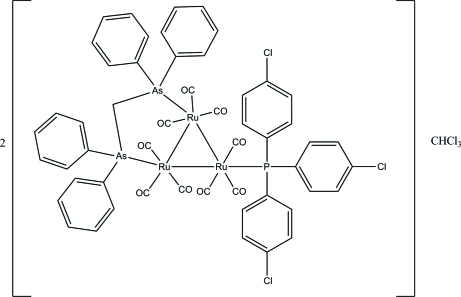


## Experimental

### 

#### Crystal data

2[Ru_3_(C_25_H_22_As_2_)(C_18_H_12_Cl_3_P)(CO)_9_]·CHCl_3_
*M* *_r_* = 2905.69
Orthorhombic, 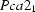

*a* = 15.4916 (6) Å
*b* = 32.0500 (11) Å
*c* = 21.1332 (8) Å
*V* = 10492.8 (7) Å^3^
*Z* = 4
Mo *K*α radiation
μ = 2.42 mm^−1^
*T* = 100 K
0.19 × 0.19 × 0.10 mm

#### Data collection

Bruker SMART APEXII CCD area-detector diffractometer
Absorption correction: multi-scan (*SADABS*; Bruker, 2005 ▶) *T*_min_ = 0.654, *T*_max_ = 0.790
73964 measured reflections
27898 independent reflections
22534 reflections with *I* > 2σ(*I*)
*R*_int_ = 0.052

#### Refinement

*R*[*F* ^2^ > 2σ(*F* ^2^)] = 0.043
*wR*(*F* ^2^) = 0.092
*S* = 1.03
27898 reflections
1298 parameters
1 restraint
H-atom parameters constrained
Δρ_max_ = 1.24 e Å^−3^
Δρ_min_ = −0.77 e Å^−3^
Absolute structure: Flack (1983 ▶), 12139 Friedel pairs
Flack parameter: 0.480 (7)

### 

Data collection: *APEX2* (Bruker, 2005 ▶); cell refinement: *SAINT* (Bruker, 2005 ▶); data reduction: *SAINT*; program(s) used to solve structure: *SHELXTL* (Sheldrick, 2008 ▶); program(s) used to refine structure: *SHELXTL*; molecular graphics: *SHELXTL*; software used to prepare material for publication: *SHELXTL* and *PLATON* (Spek, 2009 ▶).

## Supplementary Material

Crystal structure: contains datablocks global, I. DOI: 10.1107/S1600536809053884/sj2702sup1.cif

Structure factors: contains datablocks I. DOI: 10.1107/S1600536809053884/sj2702Isup2.hkl

Additional supplementary materials:  crystallographic information; 3D view; checkCIF report

## Figures and Tables

**Table 1:** Hydrogen-bond geometry (Å, °)

*D*—H⋯*A*	*D*—H	H⋯*A*	*D*⋯*A*	*D*—H⋯*A*
C5*B*—H5*BA*⋯O4*B*^i^	0.93	2.53	3.293 (8)	139
C23*B*—H23*B*⋯Cl1*B*^ii^	0.93	2.81	3.566 (7)	139
C40*B*—H40*B*⋯O3*A*^ii^	0.93	2.49	3.047 (8)	119
C4*A*—H4*AA*⋯*Cg*1^iii^	0.93	2.86	3.560 (7)	133
C4*B*—H4*BA*⋯*Cg*2^iv^	0.93	2.68	3.314 (7)	126
C16*A*—H16*A*⋯*Cg*3^v^	0.93	2.85	3.629 (7)	142
C16*B*—H16*B*⋯*Cg*2^vi^	0.93	2.94	3.591 (7)	128
C24*A*—H24*A*⋯*Cg*1^vii^	0.93	2.90	3.582 (7)	131

Symmetry codes: (i) 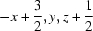; (ii) 
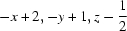
; (iii) 
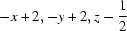
; (iv) 
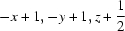
; (v) 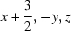; (vi) 
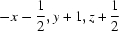
; (vii) 
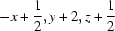
. *Cg*1 *Cg*2 and *Cg*3 are the centroids of the C14*A*–C19*A*, C20*B*–C25*B* and C1*A*–C6*A* benzene rings, respectively.

**Fig. 1.**
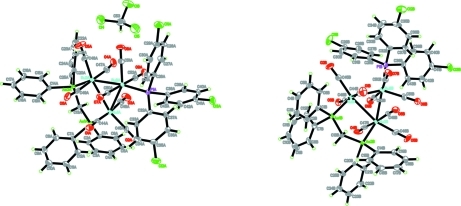
The molecular structure of the title compound with 50% probability ellipsoids for non-H atoms.

**Fig. 2.**
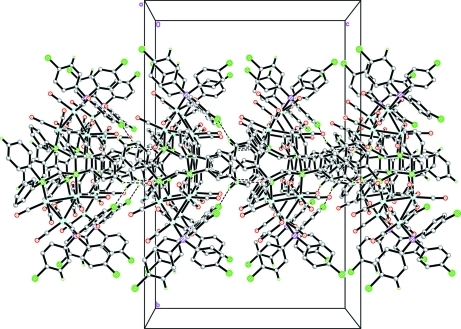
The crystal packing of the title compound, viewed down the a axis, showing the molecules linked into 3-D framework. Hydrogen atoms that not involved in the hydrogen-bonding (dashed lines) and the solvent molecules have been omitted for clarity.

## supplementary crystallographic information

### Comment

*Triangulo*-triruthenium clusters are known for their interesting structural variations and related catalytic activity. A large number of substituted derivatives, Ru_3_(CO)_12-n_*L*_n_ (*L* = group 15 ligand) have been reported (Bruce *et al.*, 1985, 1988*a*,*b*). As part of our study on the substitution of transition metal-carbonyl clusters with mixed-ligand complexes, we have published several structures of *triangulo*-triruthenium-carbonyl clusters containing mixed P/As and P/Sb ligands (Shawkataly *et al.*, 1998, 2004, 2009). Herein we report the synthesis and structure of title compound.

The asymmetry unit consists of two molecules of *triangulo*-triruthenium complex (*A* and *B*) and one molecule of chloroform solvent (Fig. 1). The bond lengths and angles of title compound are comparable to its related structure (Shawkataly *et al.*, 2009). The bis(diphenylarsino)methane ligand bridges the Ru1—Ru2 bond and the monodentate phosphine ligand bonds to the Ru3 atom. Both the phosphine and arsine ligands are equatorial with respect to the Ru_3_ triangle. Additionally, each Ru atom carries one equatorial and two axial terminal carbonyl ligands. The three phosphine-substituted benzene rings make dihedral angles (C26–C31/C32–C37, C26–C31/C38–C43 and C32–C37/C38–C43) of 73.5 (3), 57.2 (3) and 75.7 (3)° with each other in molecule *A* whereas these angles are 60.7 (3), 86.8 (3) and 54.9 (3)° in molecule *B*. The dihedral angles between the two benzene rings (C1–C6/C7–C12 and C14–C19/C20–C25) are 87.3 (3) and 89.6 (3)° for the two diphenylarsino groups in molecule *A* whereas these angles are 85.6 (3) and 87.7 (3)° in molecule *B*. In the crystal packing, the molecules are linked together into three-dimensional framework *via* intermolecular C5B—H5BA···O4B, C40B—H40B···O3A and C23B—H23B···Cl1B hydrogen bonds (Fig 2). Weak intermolecular C—H···π interactions furture stabilize the crystal structure (Table 1).

### Experimental

All manipulations were performed under a dry oxygen-free dinitrogen atmosphere using standard Schlenk techniques, all solvents were dried over sodium and distilled from sodium benzophenone ketyl under nitrogen. Tris(4-chlorophenyl)diphenylphosphine (Maybridge) was used as received and bis(diphenylarsino)methanedecacarbonyltriruthenium(0) (Bruce *et al.*, 1983) was prepared by a reported procedure. The title compound was obtained by refluxing equimolar quantities of Ru_3_(CO)_10_(µ-Ph_2_AsCH_2_AsPh_2_) (105.5 mg, 0.1 mmol) and tris(4-chlorophenyl)phosphine (36.56 mg, 0.1 mmol) in hexane under a nitrogen atmosphere. Crystals suitable for X-ray diffraction were grown by slow solvent / solvent diffusion of C_6_H_14_ into CH_2_Cl_2_.

### Refinement

All hydrogen atoms were positioned geometrically and refined using a riding model with C—H = 0.93–0.97 Å and *U*_iso_(H) = 1.2 *U*_eq_(C). The crystal studied is an inversion twin with the refined ratio of twin components being 0.480 (7):0.520 (7).

### Crystal data

**Table:**

2[Ru_3_(C_25_H_22_As_2_)(C_18_H_12_Cl_3_P)(CO)_9_]·CHCl_3_	*F*(000) = 5688
*M**_r_* = 2905.69	*D*_x_ = 1.839 Mg m^−^^3^
Orthorhombic, *P**c**a*2_1_	Mo *K*α radiation, λ = 0.71073 Å
Hall symbol: P 2c -2ac	Cell parameters from 9880 reflections
*a* = 15.4916 (6) Å	θ = 2.4–28.9°
*b* = 32.0500 (11) Å	µ = 2.42 mm^−^^1^
*c* = 21.1332 (8) Å	*T* = 100 K
*V* = 10492.8 (7) Å^3^	Block, red
*Z* = 4	0.19 × 0.19 × 0.10 mm

### Data collection

**Table:**

Bruker SMART APEXII CCD area-detector diffractometer	27898 independent reflections
Radiation source: fine-focus sealed tube	22534 reflections with *I* > 2σ(*I*)
graphite	*R*_int_ = 0.052
φ and ω scans	θ_max_ = 30.1°, θ_min_ = 1.8°
Absorption correction: multi-scan (*SADABS*; Bruker, 2005)	*h* = −21→21
*T*_min_ = 0.654, *T*_max_ = 0.790	*k* = −43→45
73964 measured reflections	*l* = −25→29

### Refinement

**Table:**

Refinement on *F*^2^	Secondary atom site location: difference Fourier map
Least-squares matrix: full	Hydrogen site location: inferred from neighbouring sites
*R*[*F*^2^ > 2σ(*F*^2^)] = 0.043	H-atom parameters constrained
*wR*(*F*^2^) = 0.092	*w* = 1/[σ^2^(*F*_o_^2^) + (0.040*P*)^2^ + 3.8533*P*] where *P* = (*F*_o_^2^ + 2*F*_c_^2^)/3
*S* = 1.03	(Δ/σ)_max_ = 0.001
27898 reflections	Δρ_max_ = 1.24 e Å^−^^3^
1298 parameters	Δρ_min_ = −0.77 e Å^−^^3^
1 restraint	Absolute structure: Flack (1983), 12139 Friedel pairs
Primary atom site location: structure-invariant direct methods	Flack parameter: 0.480 (7)

### Special details

**Table:**

Experimental. The crystal was placed in the cold stream of an Oxford Cyrosystems Cobra open-flow nitrogen cryostat (Cosier & Glazer, 1986) operating at 100.0 (1) K.
Geometry. All e.s.d.'s (except the e.s.d. in the dihedral angle between two l.s. planes) are estimated using the full covariance matrix. The cell e.s.d.'s are taken into account individually in the estimation of e.s.d.'s in distances, angles and torsion angles; correlations between e.s.d.'s in cell parameters are only used when they are defined by crystal symmetry. An approximate (isotropic) treatment of cell e.s.d.'s is used for estimating e.s.d.'s involving l.s. planes.
Refinement. Refinement of *F*^2^ against ALL reflections. The weighted *R*-factor *wR* and goodness of fit *S* are based on *F*^2^, conventional *R*-factors *R* are based on *F*, with *F* set to zero for negative *F*^2^. The threshold expression of *F*^2^ > σ(*F*^2^) is used only for calculating *R*-factors(gt) *etc*. and is not relevant to the choice of reflections for refinement. *R*-factors based on *F*^2^ are statistically about twice as large as those based on *F*, and *R*- factors based on ALL data will be even larger.

### Fractional atomic coordinates and isotropic or equivalent isotropic displacement parameters (Å^2^)

**Table:**

	*x*	*y*	*z*	*U*_iso_*/*U*_eq_	
Ru1A	0.71961 (3)	0.902795 (13)	0.74015 (2)	0.01573 (9)	
Ru2A	0.76030 (3)	0.889859 (13)	0.87170 (2)	0.01597 (9)	
Ru3A	0.66115 (3)	0.829574 (13)	0.80543 (2)	0.01663 (9)	
As1A	0.79716 (3)	0.968408 (16)	0.73567 (3)	0.01486 (11)	
As2A	0.86050 (3)	0.947998 (17)	0.87988 (3)	0.01465 (11)	
Cl1A	0.43461 (12)	0.65412 (5)	0.93450 (8)	0.0421 (4)	
Cl2A	0.25032 (11)	0.84215 (5)	0.58560 (9)	0.0397 (4)	
Cl3A	0.79590 (12)	0.69040 (5)	0.52357 (8)	0.0402 (4)	
P1A	0.58346 (9)	0.78529 (4)	0.73821 (7)	0.0184 (3)	
O1A	0.5473 (3)	0.94607 (14)	0.7690 (2)	0.0298 (10)	
O2A	0.6491 (3)	0.88986 (16)	0.6077 (2)	0.0400 (12)	
O3A	0.8980 (3)	0.86474 (16)	0.7167 (3)	0.0479 (14)	
O4A	0.9222 (3)	0.83606 (14)	0.8517 (2)	0.0382 (12)	
O5A	0.7450 (4)	0.85500 (17)	1.0054 (2)	0.0487 (14)	
O6A	0.6039 (3)	0.94517 (15)	0.9014 (2)	0.0343 (11)	
O7A	0.4926 (3)	0.87316 (13)	0.8457 (2)	0.0269 (9)	
O8A	0.6578 (3)	0.77439 (13)	0.9213 (2)	0.0321 (10)	
O9A	0.8254 (3)	0.78334 (13)	0.7642 (2)	0.0339 (10)	
C1A	0.8675 (4)	0.98065 (18)	0.6608 (3)	0.0190 (12)	
C2A	0.9114 (4)	1.01815 (18)	0.6577 (3)	0.0210 (12)	
H2AA	0.9063	1.0376	0.6901	0.025*	
C3A	0.9635 (4)	1.0265 (2)	0.6054 (3)	0.0269 (14)	
H3AA	0.9933	1.0517	0.6031	0.032*	
C4A	0.9709 (4)	0.99839 (19)	0.5582 (3)	0.0286 (14)	
H4AA	1.0049	1.0046	0.5232	0.034*	
C5A	0.9286 (4)	0.96040 (19)	0.5614 (3)	0.0254 (13)	
H5AA	0.9353	0.9409	0.5291	0.030*	
C6A	0.8760 (4)	0.95147 (19)	0.6130 (3)	0.0223 (12)	
H6AA	0.8468	0.9261	0.6153	0.027*	
C7A	0.7301 (3)	1.01969 (17)	0.7408 (3)	0.0180 (11)	
C8A	0.7528 (4)	1.05358 (18)	0.7793 (3)	0.0270 (13)	
H8AA	0.8005	1.0520	0.8059	0.032*	
C9A	0.7030 (5)	1.0894 (2)	0.7769 (3)	0.0368 (17)	
H9AA	0.7182	1.1120	0.8020	0.044*	
C10A	0.6322 (5)	1.0923 (2)	0.7386 (4)	0.0391 (17)	
H10A	0.5999	1.1167	0.7378	0.047*	
C11A	0.6084 (4)	1.05847 (19)	0.7008 (3)	0.0285 (14)	
H11A	0.5601	1.0601	0.6748	0.034*	
C12A	0.6583 (4)	1.02217 (19)	0.7026 (3)	0.0241 (13)	
H12A	0.6428	0.9994	0.6777	0.029*	
C13A	0.8905 (4)	0.97306 (17)	0.7977 (3)	0.0189 (11)	
H13A	0.9042	1.0023	0.8039	0.023*	
H13B	0.9416	0.9594	0.7811	0.023*	
C14A	0.8387 (4)	0.99512 (17)	0.9355 (3)	0.0194 (12)	
C15A	0.7887 (4)	0.98787 (18)	0.9887 (3)	0.0218 (12)	
H15A	0.7599	0.9626	0.9936	0.026*	
C16A	0.7821 (4)	1.0185 (2)	1.0343 (3)	0.0294 (14)	
H16A	0.7489	1.0137	1.0702	0.035*	
C17A	0.8239 (4)	1.0565 (2)	1.0274 (3)	0.0292 (14)	
H17A	0.8196	1.0768	1.0586	0.035*	
C18A	0.8718 (4)	1.06361 (19)	0.9739 (3)	0.0266 (14)	
H18A	0.8990	1.0892	0.9686	0.032*	
C19A	0.8803 (4)	1.03319 (18)	0.9274 (3)	0.0223 (12)	
H19A	0.9134	1.0382	0.8914	0.027*	
C20A	0.9736 (3)	0.93291 (17)	0.9125 (3)	0.0187 (11)	
C21A	0.9755 (4)	0.90174 (17)	0.9605 (3)	0.0247 (13)	
H21A	0.9247	0.8894	0.9746	0.030*	
C22A	1.0539 (4)	0.8904 (2)	0.9856 (3)	0.0358 (16)	
H22A	1.0553	0.8701	1.0170	0.043*	
C23A	1.1295 (4)	0.9077 (2)	0.9661 (3)	0.0361 (16)	
H23A	1.1818	0.8987	0.9829	0.043*	
C24A	1.1274 (4)	0.9389 (2)	0.9212 (4)	0.0417 (19)	
H24A	1.1785	0.9518	0.9090	0.050*	
C25A	1.0485 (4)	0.9515 (2)	0.8937 (3)	0.0284 (14)	
H25A	1.0476	0.9723	0.8630	0.034*	
C26A	0.5353 (4)	0.74395 (16)	0.7869 (2)	0.0194 (11)	
C27A	0.5848 (4)	0.71001 (17)	0.8077 (3)	0.0274 (13)	
H27A	0.6397	0.7061	0.7909	0.033*	
C28A	0.5546 (4)	0.68241 (18)	0.8520 (3)	0.0285 (13)	
H28A	0.5880	0.6598	0.8645	0.034*	
C29A	0.4731 (4)	0.68876 (18)	0.8778 (3)	0.0257 (12)	
C30A	0.4237 (4)	0.72151 (19)	0.8587 (3)	0.0288 (13)	
H30A	0.3688	0.7253	0.8756	0.035*	
C31A	0.4554 (4)	0.74938 (18)	0.8139 (3)	0.0273 (13)	
H31A	0.4218	0.7721	0.8020	0.033*	
C32A	0.4898 (3)	0.80376 (16)	0.6923 (2)	0.0188 (11)	
C33A	0.4661 (4)	0.84513 (16)	0.6901 (3)	0.0224 (12)	
H33A	0.4999	0.8649	0.7106	0.027*	
C34A	0.3927 (5)	0.8577 (2)	0.6579 (3)	0.0338 (15)	
H34A	0.3757	0.8856	0.6581	0.041*	
C35A	0.3450 (4)	0.82813 (18)	0.6256 (3)	0.0268 (13)	
C36A	0.3686 (4)	0.78665 (16)	0.6257 (3)	0.0225 (12)	
H36A	0.3360	0.7671	0.6036	0.027*	
C37A	0.4406 (3)	0.77448 (16)	0.6586 (3)	0.0223 (12)	
H37A	0.4569	0.7466	0.6587	0.027*	
C38A	0.6430 (4)	0.75671 (16)	0.6761 (3)	0.0190 (11)	
C39A	0.6216 (4)	0.71621 (16)	0.6575 (3)	0.0233 (12)	
H39A	0.5766	0.7023	0.6776	0.028*	
C40A	0.6665 (4)	0.69638 (17)	0.6094 (3)	0.0259 (13)	
H40A	0.6509	0.6696	0.5968	0.031*	
C41A	0.7348 (4)	0.71658 (17)	0.5801 (3)	0.0279 (13)	
C42A	0.7567 (4)	0.75708 (18)	0.5968 (3)	0.0294 (13)	
H42A	0.8017	0.7708	0.5764	0.035*	
C43A	0.7105 (4)	0.77692 (16)	0.6446 (3)	0.0245 (12)	
H43A	0.7248	0.8041	0.6559	0.029*	
C44A	0.6112 (3)	0.92893 (17)	0.7615 (3)	0.0169 (11)	
C45A	0.6775 (4)	0.89446 (19)	0.6566 (3)	0.0258 (13)	
C46A	0.8294 (4)	0.87641 (19)	0.7277 (3)	0.0268 (14)	
C47A	0.8602 (4)	0.85520 (19)	0.8551 (3)	0.0264 (14)	
C48A	0.7537 (4)	0.86776 (18)	0.9554 (3)	0.0244 (13)	
C49A	0.6608 (4)	0.92373 (19)	0.8869 (3)	0.0225 (12)	
C50A	0.5572 (4)	0.85929 (17)	0.8307 (3)	0.0209 (12)	
C51A	0.6575 (3)	0.79496 (17)	0.8782 (3)	0.0224 (12)	
C52A	0.7664 (4)	0.80307 (18)	0.7795 (3)	0.0240 (13)	
Ru1B	0.76562 (3)	0.404684 (13)	0.21133 (2)	0.01615 (9)	
Ru2B	0.71762 (3)	0.390935 (14)	0.08150 (2)	0.01571 (9)	
Ru3B	0.82420 (3)	0.331820 (13)	0.14264 (2)	0.01630 (9)	
As1B	0.69289 (3)	0.471718 (17)	0.21422 (3)	0.01617 (11)	
As2B	0.61823 (3)	0.448989 (17)	0.07413 (3)	0.01516 (11)	
Cl1B	1.23194 (11)	0.36598 (7)	0.33938 (9)	0.0486 (5)	
Cl2B	0.70877 (12)	0.17846 (6)	0.39212 (9)	0.0480 (5)	
Cl3B	1.03218 (12)	0.15085 (5)	−0.00982 (8)	0.0367 (4)	
P1B	0.91864 (9)	0.28571 (5)	0.19197 (7)	0.0193 (3)	
O1B	0.5867 (3)	0.37206 (15)	0.2505 (2)	0.0379 (12)	
O2B	0.8387 (3)	0.40260 (16)	0.3447 (2)	0.0404 (12)	
O3B	0.9418 (3)	0.44198 (13)	0.1757 (2)	0.0288 (10)	
O4B	0.8718 (3)	0.44566 (15)	0.0440 (2)	0.0350 (11)	
O5B	0.7101 (3)	0.35180 (16)	−0.0490 (2)	0.0439 (13)	
O6B	0.5676 (3)	0.33425 (13)	0.1214 (2)	0.0340 (11)	
O7B	0.6812 (3)	0.29361 (12)	0.2249 (2)	0.0312 (10)	
O8B	0.7804 (3)	0.27596 (12)	0.0315 (2)	0.0344 (10)	
O9B	0.9868 (3)	0.37323 (13)	0.0875 (2)	0.0298 (10)	
C1B	0.6292 (3)	0.48489 (19)	0.2911 (3)	0.0202 (12)	
C2B	0.5829 (4)	0.52199 (19)	0.2950 (3)	0.0287 (14)	
H2BA	0.5841	0.5409	0.2616	0.034*	
C3B	0.5350 (5)	0.5308 (2)	0.3490 (3)	0.0315 (15)	
H3BA	0.5031	0.5553	0.3514	0.038*	
C4B	0.5346 (4)	0.5034 (2)	0.3989 (3)	0.0322 (16)	
H4BA	0.5031	0.5095	0.4352	0.039*	
C5B	0.5810 (4)	0.4665 (2)	0.3954 (3)	0.0269 (14)	
H5BA	0.5808	0.4481	0.4294	0.032*	
C6B	0.6275 (4)	0.4571 (2)	0.3414 (3)	0.0245 (13)	
H6BA	0.6577	0.4321	0.3389	0.029*	
C7B	0.7590 (4)	0.52269 (18)	0.2058 (3)	0.0219 (12)	
C8B	0.7296 (4)	0.55764 (19)	0.1754 (3)	0.0303 (15)	
H8BA	0.6770	0.5566	0.1543	0.036*	
C9B	0.7772 (5)	0.5950 (2)	0.1753 (4)	0.0412 (18)	
H9BA	0.7574	0.6184	0.1537	0.049*	
C10B	0.8549 (5)	0.5958 (2)	0.2083 (4)	0.0389 (17)	
H10B	0.8868	0.6204	0.2096	0.047*	
C11B	0.8848 (4)	0.5614 (2)	0.2388 (3)	0.0360 (16)	
H11B	0.9371	0.5626	0.2603	0.043*	
C12B	0.8379 (4)	0.52451 (19)	0.2380 (3)	0.0259 (13)	
H12B	0.8588	0.5010	0.2589	0.031*	
C13B	0.5951 (3)	0.47638 (18)	0.1548 (2)	0.0184 (11)	
H13C	0.5442	0.4640	0.1738	0.022*	
H13D	0.5828	0.5056	0.1472	0.022*	
C14B	0.5029 (4)	0.43323 (18)	0.0468 (3)	0.0202 (12)	
C15B	0.4308 (4)	0.4566 (2)	0.0619 (3)	0.0253 (13)	
H15B	0.4362	0.4798	0.0880	0.030*	
C16B	0.3502 (4)	0.4457 (2)	0.0382 (3)	0.0330 (15)	
H16B	0.3026	0.4625	0.0468	0.040*	
C17B	0.3405 (4)	0.4113 (2)	0.0032 (3)	0.0357 (16)	
H17B	0.2858	0.4037	−0.0107	0.043*	
C18B	0.4118 (4)	0.3865 (2)	−0.0129 (3)	0.0319 (14)	
H18B	0.4050	0.3626	−0.0373	0.038*	
C19B	0.4932 (4)	0.39849 (18)	0.0085 (3)	0.0253 (13)	
H19B	0.5415	0.3830	−0.0030	0.030*	
C20B	0.6397 (3)	0.49514 (18)	0.0165 (3)	0.0176 (11)	
C21B	0.6873 (4)	0.48708 (19)	−0.0379 (3)	0.0232 (12)	
H21B	0.7145	0.4615	−0.0434	0.028*	
C22B	0.6938 (4)	0.5183 (2)	−0.0849 (3)	0.0300 (14)	
H22B	0.7263	0.5136	−0.1212	0.036*	
C23B	0.6513 (4)	0.55585 (19)	−0.0764 (3)	0.0279 (14)	
H23B	0.6528	0.5758	−0.1084	0.034*	
C24B	0.6069 (4)	0.56427 (19)	−0.0217 (3)	0.0254 (13)	
H24B	0.5823	0.5904	−0.0156	0.030*	
C25B	0.5986 (4)	0.53407 (17)	0.0241 (3)	0.0225 (12)	
H25B	0.5660	0.5393	0.0602	0.027*	
C26B	1.0114 (4)	0.30885 (16)	0.2309 (3)	0.0222 (12)	
C27B	1.0950 (4)	0.29864 (18)	0.2171 (3)	0.0269 (12)	
H27B	1.1059	0.2789	0.1858	0.032*	
C28B	1.1641 (4)	0.31693 (19)	0.2486 (3)	0.0335 (14)	
H28B	1.2206	0.3100	0.2382	0.040*	
C29B	1.1470 (4)	0.3453 (2)	0.2951 (3)	0.0304 (14)	
C30B	1.0650 (4)	0.35748 (19)	0.3093 (3)	0.0332 (15)	
H30B	1.0548	0.3776	0.3402	0.040*	
C31B	0.9973 (4)	0.3392 (2)	0.2768 (3)	0.0352 (16)	
H31B	0.9411	0.3475	0.2858	0.042*	
C32B	0.8686 (4)	0.25350 (18)	0.2524 (3)	0.0239 (13)	
C33B	0.8762 (5)	0.2596 (2)	0.3172 (3)	0.0425 (18)	
H33B	0.9137	0.2799	0.3325	0.051*	
C34B	0.8283 (5)	0.2357 (3)	0.3597 (3)	0.049 (2)	
H34B	0.8355	0.2397	0.4030	0.059*	
C35B	0.7712 (4)	0.2068 (2)	0.3386 (3)	0.0373 (16)	
C36B	0.7611 (4)	0.19962 (18)	0.2742 (3)	0.0298 (13)	
H36B	0.7223	0.1797	0.2597	0.036*	
C37B	0.8098 (4)	0.22274 (17)	0.2322 (3)	0.0286 (13)	
H37B	0.8035	0.2179	0.1891	0.034*	
C38B	0.9646 (4)	0.24721 (17)	0.1374 (3)	0.0208 (11)	
C39B	0.9812 (4)	0.20657 (19)	0.1534 (3)	0.0331 (14)	
H39B	0.9776	0.1988	0.1957	0.040*	
C40B	1.0030 (4)	0.17686 (19)	0.1093 (3)	0.0329 (15)	
H40B	1.0125	0.1494	0.1215	0.039*	
C41B	1.0105 (4)	0.18817 (18)	0.0477 (3)	0.0273 (14)	
C42B	1.0025 (4)	0.22937 (18)	0.0293 (3)	0.0300 (13)	
H42B	1.0131	0.2371	−0.0124	0.036*	
C43B	0.9788 (4)	0.25862 (17)	0.0732 (3)	0.0260 (12)	
H43B	0.9719	0.2863	0.0609	0.031*	
C44B	0.6542 (4)	0.38193 (18)	0.2335 (3)	0.0244 (12)	
C45B	0.8092 (4)	0.40198 (19)	0.2940 (3)	0.0270 (13)	
C46B	0.8754 (4)	0.42610 (18)	0.1845 (3)	0.0209 (12)	
C47B	0.8165 (4)	0.42541 (19)	0.0607 (3)	0.0241 (13)	
C48B	0.7119 (4)	0.3673 (2)	0.0001 (3)	0.0288 (14)	
C49B	0.6249 (4)	0.35482 (18)	0.1088 (3)	0.0210 (12)	
C50B	0.7320 (4)	0.31035 (18)	0.1952 (3)	0.0227 (12)	
C51B	0.7993 (4)	0.29710 (17)	0.0731 (3)	0.0247 (12)	
C52B	0.9240 (4)	0.35981 (18)	0.1074 (3)	0.0217 (12)	
Cl4	0.92574 (16)	0.79197 (6)	1.01220 (10)	0.0600 (6)	
Cl5	0.87858 (17)	0.73493 (7)	0.91326 (12)	0.0761 (8)	
Cl6	1.03351 (14)	0.72054 (7)	0.98448 (11)	0.0591 (6)	
C53	0.9641 (5)	0.7592 (2)	0.9540 (3)	0.0466 (19)	
H53A	0.9968	0.7760	0.9236	0.056*	

### Atomic displacement parameters (Å^2^)

**Table:**

	*U*^11^	*U*^22^	*U*^33^	*U*^12^	*U*^13^	*U*^23^
Ru1A	0.0220 (2)	0.0141 (2)	0.0111 (2)	−0.00134 (15)	−0.00042 (17)	0.00084 (18)
Ru2A	0.0187 (2)	0.0162 (2)	0.0130 (2)	−0.00366 (16)	−0.00208 (17)	0.00232 (17)
Ru3A	0.0217 (2)	0.0133 (2)	0.0148 (2)	−0.00346 (16)	−0.00155 (17)	0.00089 (18)
As1A	0.0200 (3)	0.0148 (3)	0.0098 (3)	−0.0013 (2)	0.0006 (2)	0.0013 (2)
As2A	0.0175 (2)	0.0157 (3)	0.0107 (3)	−0.0025 (2)	−0.0010 (2)	0.0005 (2)
Cl1A	0.0538 (11)	0.0366 (9)	0.0359 (9)	0.0005 (8)	0.0132 (8)	0.0160 (7)
Cl2A	0.0397 (9)	0.0349 (8)	0.0445 (10)	0.0019 (7)	−0.0166 (8)	0.0117 (7)
Cl3A	0.0551 (11)	0.0297 (8)	0.0357 (9)	0.0026 (7)	0.0142 (8)	−0.0075 (7)
P1A	0.0237 (7)	0.0138 (6)	0.0178 (7)	−0.0018 (5)	−0.0041 (6)	0.0004 (6)
O1A	0.024 (2)	0.035 (3)	0.030 (2)	0.0037 (19)	−0.0022 (18)	0.006 (2)
O2A	0.052 (3)	0.052 (3)	0.016 (2)	0.000 (2)	−0.006 (2)	−0.010 (2)
O3A	0.048 (3)	0.045 (3)	0.051 (3)	0.020 (2)	0.023 (3)	0.013 (3)
O4A	0.035 (3)	0.030 (2)	0.050 (3)	0.004 (2)	−0.009 (2)	−0.014 (2)
O5A	0.054 (3)	0.072 (4)	0.021 (3)	−0.024 (3)	−0.007 (2)	0.018 (2)
O6A	0.031 (2)	0.048 (3)	0.023 (2)	0.004 (2)	−0.0027 (19)	−0.012 (2)
O7A	0.027 (2)	0.026 (2)	0.028 (2)	0.0024 (17)	0.0017 (18)	0.0024 (18)
O8A	0.040 (3)	0.032 (2)	0.024 (2)	−0.0026 (19)	−0.0049 (19)	0.0146 (19)
O9A	0.033 (2)	0.028 (2)	0.040 (3)	0.0027 (19)	−0.002 (2)	−0.011 (2)
C1A	0.025 (3)	0.019 (3)	0.013 (3)	0.004 (2)	0.005 (2)	0.004 (2)
C2A	0.031 (3)	0.018 (3)	0.014 (3)	−0.004 (2)	−0.003 (2)	0.001 (2)
C3A	0.034 (3)	0.025 (3)	0.021 (3)	−0.001 (3)	0.001 (3)	0.010 (3)
C4A	0.032 (3)	0.030 (3)	0.024 (3)	0.001 (3)	0.011 (3)	0.009 (3)
C5A	0.036 (3)	0.027 (3)	0.013 (3)	0.011 (2)	0.004 (2)	−0.001 (2)
C6A	0.026 (3)	0.024 (3)	0.017 (3)	−0.001 (2)	0.000 (2)	0.004 (2)
C7A	0.027 (3)	0.011 (2)	0.015 (3)	0.0003 (19)	0.009 (2)	0.003 (2)
C8A	0.029 (3)	0.025 (3)	0.027 (3)	0.003 (3)	−0.003 (3)	−0.002 (3)
C9A	0.061 (5)	0.018 (3)	0.031 (4)	0.001 (3)	−0.002 (3)	−0.004 (3)
C10A	0.052 (4)	0.029 (4)	0.036 (4)	0.014 (3)	0.004 (4)	0.003 (3)
C11A	0.030 (3)	0.024 (3)	0.031 (4)	0.007 (2)	−0.001 (3)	0.006 (3)
C12A	0.034 (3)	0.021 (3)	0.017 (3)	0.001 (2)	0.004 (2)	0.004 (2)
C13A	0.022 (3)	0.021 (3)	0.013 (3)	−0.004 (2)	−0.001 (2)	0.002 (2)
C14A	0.024 (3)	0.016 (3)	0.018 (3)	−0.003 (2)	−0.007 (2)	0.002 (2)
C15A	0.028 (3)	0.024 (3)	0.014 (3)	0.000 (2)	0.003 (2)	−0.004 (2)
C16A	0.029 (3)	0.038 (4)	0.021 (3)	0.005 (3)	−0.003 (3)	−0.011 (3)
C17A	0.034 (3)	0.028 (3)	0.025 (3)	0.007 (3)	−0.007 (3)	−0.009 (3)
C18A	0.035 (3)	0.016 (3)	0.029 (4)	0.003 (2)	−0.009 (3)	−0.009 (3)
C19A	0.023 (3)	0.026 (3)	0.017 (3)	−0.003 (2)	−0.001 (2)	−0.002 (2)
C20A	0.017 (3)	0.020 (3)	0.018 (3)	−0.001 (2)	−0.001 (2)	−0.005 (2)
C21A	0.026 (3)	0.023 (3)	0.025 (3)	0.000 (2)	−0.009 (2)	0.002 (2)
C22A	0.044 (4)	0.026 (3)	0.037 (4)	−0.003 (3)	−0.023 (3)	0.001 (3)
C23A	0.025 (3)	0.049 (4)	0.034 (4)	0.007 (3)	−0.010 (3)	−0.003 (3)
C24A	0.027 (3)	0.062 (5)	0.037 (4)	−0.013 (3)	−0.005 (3)	−0.011 (4)
C25A	0.028 (3)	0.033 (3)	0.025 (3)	−0.004 (3)	−0.004 (3)	−0.002 (3)
C26A	0.025 (3)	0.018 (3)	0.015 (3)	−0.006 (2)	−0.003 (2)	−0.001 (2)
C27A	0.027 (3)	0.023 (3)	0.032 (3)	−0.002 (2)	0.007 (3)	0.006 (3)
C28A	0.036 (3)	0.024 (3)	0.026 (3)	0.000 (3)	−0.001 (3)	0.011 (2)
C29A	0.032 (3)	0.023 (3)	0.022 (3)	−0.004 (2)	−0.001 (3)	0.007 (2)
C30A	0.026 (3)	0.038 (3)	0.022 (3)	0.001 (3)	0.004 (2)	0.001 (3)
C31A	0.035 (3)	0.023 (3)	0.024 (3)	−0.001 (2)	0.000 (3)	0.003 (2)
C32A	0.022 (3)	0.020 (3)	0.015 (3)	0.002 (2)	−0.002 (2)	−0.001 (2)
C33A	0.035 (3)	0.015 (3)	0.017 (3)	−0.006 (2)	−0.004 (2)	−0.003 (2)
C34A	0.045 (4)	0.024 (3)	0.033 (4)	0.008 (3)	−0.002 (3)	0.007 (3)
C35A	0.029 (3)	0.026 (3)	0.026 (3)	0.001 (2)	−0.006 (2)	0.008 (2)
C36A	0.025 (3)	0.020 (3)	0.022 (3)	−0.002 (2)	−0.005 (2)	−0.004 (2)
C37A	0.022 (3)	0.015 (2)	0.030 (3)	−0.003 (2)	−0.006 (2)	−0.003 (2)
C38A	0.025 (3)	0.012 (2)	0.020 (3)	0.003 (2)	−0.001 (2)	0.000 (2)
C39A	0.028 (3)	0.018 (3)	0.024 (3)	−0.003 (2)	−0.007 (2)	0.000 (2)
C40A	0.039 (3)	0.014 (3)	0.025 (3)	0.002 (2)	−0.004 (3)	−0.006 (2)
C41A	0.043 (4)	0.021 (3)	0.020 (3)	0.002 (2)	0.000 (3)	−0.002 (2)
C42A	0.033 (3)	0.024 (3)	0.030 (3)	0.004 (3)	0.006 (3)	0.002 (2)
C43A	0.038 (3)	0.012 (2)	0.023 (3)	−0.002 (2)	−0.003 (3)	−0.002 (2)
C44A	0.020 (3)	0.020 (3)	0.011 (3)	−0.003 (2)	−0.003 (2)	0.003 (2)
C45A	0.039 (3)	0.019 (3)	0.020 (3)	0.002 (2)	0.002 (3)	−0.009 (2)
C46A	0.032 (3)	0.022 (3)	0.026 (3)	0.005 (2)	0.007 (3)	0.005 (2)
C47A	0.026 (3)	0.020 (3)	0.033 (4)	−0.002 (2)	−0.008 (3)	−0.001 (3)
C48A	0.028 (3)	0.028 (3)	0.017 (3)	−0.016 (3)	−0.005 (2)	0.003 (2)
C49A	0.026 (3)	0.029 (3)	0.012 (3)	−0.001 (2)	−0.007 (2)	−0.006 (2)
C50A	0.032 (3)	0.012 (3)	0.019 (3)	−0.001 (2)	−0.004 (2)	0.001 (2)
C51A	0.025 (3)	0.019 (3)	0.023 (3)	−0.002 (2)	0.002 (2)	0.004 (2)
C52A	0.025 (3)	0.024 (3)	0.023 (3)	−0.009 (2)	−0.002 (2)	−0.002 (2)
Ru1B	0.0216 (2)	0.0152 (2)	0.0117 (2)	0.00039 (16)	0.00004 (17)	0.00054 (18)
Ru2B	0.0201 (2)	0.0162 (2)	0.0108 (2)	0.00123 (16)	0.00079 (16)	0.00046 (17)
Ru3B	0.0188 (2)	0.0151 (2)	0.0151 (2)	0.00133 (16)	0.00044 (17)	0.00062 (18)
As1B	0.0215 (3)	0.0158 (3)	0.0112 (3)	−0.0002 (2)	0.0019 (2)	−0.0004 (2)
As2B	0.0191 (3)	0.0155 (3)	0.0109 (3)	0.0010 (2)	0.0008 (2)	0.0007 (2)
Cl1B	0.0334 (9)	0.0691 (12)	0.0433 (10)	−0.0160 (8)	−0.0034 (8)	−0.0224 (9)
Cl2B	0.0498 (11)	0.0529 (11)	0.0411 (10)	−0.0019 (8)	0.0086 (8)	0.0171 (8)
Cl3B	0.0533 (10)	0.0245 (8)	0.0321 (9)	−0.0027 (7)	0.0119 (7)	−0.0108 (6)
P1B	0.0207 (7)	0.0173 (7)	0.0199 (7)	0.0037 (5)	−0.0028 (5)	−0.0021 (6)
O1B	0.037 (3)	0.038 (3)	0.039 (3)	−0.008 (2)	0.019 (2)	−0.003 (2)
O2B	0.051 (3)	0.052 (3)	0.018 (2)	0.008 (2)	−0.006 (2)	0.002 (2)
O3B	0.029 (2)	0.028 (2)	0.029 (2)	−0.0019 (18)	0.0006 (19)	−0.0055 (19)
O4B	0.032 (3)	0.043 (3)	0.029 (3)	−0.006 (2)	0.001 (2)	0.024 (2)
O5B	0.059 (3)	0.057 (3)	0.015 (2)	0.020 (3)	−0.009 (2)	−0.018 (2)
O6B	0.032 (2)	0.030 (2)	0.040 (3)	−0.0105 (19)	−0.014 (2)	0.011 (2)
O7B	0.032 (2)	0.024 (2)	0.038 (3)	0.0009 (17)	0.0086 (19)	0.0082 (19)
O8B	0.051 (3)	0.026 (2)	0.027 (2)	−0.002 (2)	−0.005 (2)	−0.0023 (19)
O9B	0.030 (2)	0.026 (2)	0.034 (3)	−0.0025 (17)	0.007 (2)	0.0031 (19)
C1B	0.021 (3)	0.028 (3)	0.012 (3)	−0.002 (2)	0.000 (2)	−0.003 (2)
C2B	0.036 (3)	0.026 (3)	0.025 (3)	−0.001 (3)	0.006 (3)	−0.003 (3)
C3B	0.046 (4)	0.022 (3)	0.026 (4)	0.000 (3)	0.011 (3)	−0.006 (3)
C4B	0.041 (4)	0.038 (4)	0.018 (3)	−0.009 (3)	0.010 (3)	−0.012 (3)
C5B	0.035 (3)	0.035 (4)	0.011 (3)	−0.004 (3)	0.002 (2)	0.002 (2)
C6B	0.035 (3)	0.021 (3)	0.018 (3)	−0.004 (2)	−0.003 (2)	0.004 (2)
C7B	0.025 (3)	0.023 (3)	0.018 (3)	0.004 (2)	0.004 (2)	−0.003 (2)
C8B	0.039 (4)	0.019 (3)	0.033 (4)	−0.003 (3)	0.004 (3)	−0.005 (3)
C9B	0.059 (5)	0.025 (3)	0.039 (4)	−0.001 (3)	0.014 (4)	0.007 (3)
C10B	0.055 (4)	0.024 (3)	0.038 (4)	−0.016 (3)	0.005 (4)	−0.009 (3)
C11B	0.031 (3)	0.042 (4)	0.035 (4)	−0.007 (3)	0.012 (3)	−0.014 (3)
C12B	0.033 (3)	0.022 (3)	0.023 (3)	−0.002 (2)	0.007 (3)	−0.002 (3)
C13B	0.022 (3)	0.023 (3)	0.010 (3)	−0.002 (2)	0.000 (2)	0.001 (2)
C14B	0.024 (3)	0.022 (3)	0.015 (3)	−0.005 (2)	−0.008 (2)	0.009 (2)
C15B	0.025 (3)	0.036 (3)	0.015 (3)	0.006 (2)	0.004 (2)	0.006 (2)
C16B	0.025 (3)	0.040 (4)	0.034 (4)	0.009 (3)	−0.003 (3)	0.012 (3)
C17B	0.030 (3)	0.039 (4)	0.039 (4)	−0.005 (3)	−0.008 (3)	0.016 (3)
C18B	0.036 (3)	0.025 (3)	0.035 (4)	−0.007 (3)	−0.011 (3)	0.000 (3)
C19B	0.032 (3)	0.021 (3)	0.023 (3)	0.002 (2)	−0.005 (2)	0.000 (2)
C20B	0.014 (2)	0.027 (3)	0.012 (3)	−0.003 (2)	−0.005 (2)	0.001 (2)
C21B	0.024 (3)	0.030 (3)	0.016 (3)	0.000 (2)	0.000 (2)	0.000 (2)
C22B	0.029 (3)	0.042 (4)	0.019 (3)	0.001 (3)	0.005 (3)	0.005 (3)
C23B	0.030 (3)	0.025 (3)	0.028 (3)	−0.006 (3)	−0.003 (3)	0.009 (3)
C24B	0.028 (3)	0.018 (3)	0.030 (3)	0.000 (2)	−0.001 (3)	0.003 (3)
C25B	0.022 (3)	0.020 (3)	0.025 (3)	0.000 (2)	−0.004 (2)	0.000 (2)
C26B	0.026 (3)	0.015 (3)	0.026 (3)	0.005 (2)	−0.006 (2)	−0.002 (2)
C27B	0.028 (3)	0.030 (3)	0.023 (3)	0.001 (2)	0.001 (2)	−0.004 (3)
C28B	0.028 (3)	0.032 (3)	0.041 (4)	0.006 (3)	−0.004 (3)	−0.006 (3)
C29B	0.030 (3)	0.033 (3)	0.028 (3)	−0.012 (3)	−0.003 (3)	−0.004 (3)
C30B	0.035 (4)	0.025 (3)	0.040 (4)	0.005 (3)	−0.012 (3)	−0.013 (3)
C31B	0.029 (3)	0.044 (4)	0.032 (4)	0.014 (3)	−0.011 (3)	−0.012 (3)
C32B	0.025 (3)	0.026 (3)	0.021 (3)	0.008 (2)	−0.002 (2)	0.006 (2)
C33B	0.048 (4)	0.053 (4)	0.027 (4)	−0.018 (3)	−0.012 (3)	0.010 (3)
C34B	0.048 (4)	0.076 (5)	0.023 (3)	−0.024 (4)	−0.001 (3)	−0.001 (4)
C35B	0.035 (4)	0.036 (4)	0.040 (4)	0.005 (3)	0.008 (3)	0.025 (3)
C36B	0.036 (3)	0.022 (3)	0.031 (3)	0.001 (2)	−0.007 (3)	0.003 (3)
C37B	0.040 (3)	0.021 (3)	0.025 (3)	0.000 (2)	−0.001 (3)	0.002 (2)
C38B	0.023 (3)	0.020 (3)	0.019 (3)	0.000 (2)	−0.002 (2)	−0.005 (2)
C39B	0.047 (4)	0.029 (3)	0.023 (3)	0.009 (3)	0.002 (3)	0.003 (3)
C40B	0.045 (4)	0.020 (3)	0.034 (4)	0.006 (3)	−0.003 (3)	0.003 (3)
C41B	0.023 (3)	0.022 (3)	0.037 (4)	−0.003 (2)	0.004 (3)	−0.009 (3)
C42B	0.033 (3)	0.029 (3)	0.029 (3)	0.005 (3)	0.005 (3)	−0.004 (3)
C43B	0.029 (3)	0.018 (3)	0.031 (3)	0.004 (2)	0.007 (3)	−0.001 (2)
C44B	0.032 (3)	0.019 (3)	0.022 (3)	0.000 (2)	0.003 (3)	0.001 (2)
C45B	0.036 (3)	0.025 (3)	0.020 (3)	0.004 (3)	0.001 (3)	0.000 (2)
C46B	0.024 (3)	0.019 (3)	0.020 (3)	−0.001 (2)	0.000 (2)	−0.007 (2)
C47B	0.031 (3)	0.030 (3)	0.012 (3)	0.010 (3)	0.000 (2)	0.006 (2)
C48B	0.029 (3)	0.038 (4)	0.019 (3)	0.011 (3)	−0.003 (2)	0.001 (3)
C49B	0.026 (3)	0.016 (3)	0.021 (3)	0.000 (2)	−0.007 (2)	0.001 (2)
C50B	0.027 (3)	0.022 (3)	0.019 (3)	0.000 (2)	0.000 (2)	0.002 (2)
C51B	0.029 (3)	0.019 (3)	0.026 (3)	−0.003 (2)	0.006 (3)	−0.005 (2)
C52B	0.026 (3)	0.019 (3)	0.021 (3)	0.007 (2)	0.000 (2)	−0.002 (2)
Cl4	0.0835 (16)	0.0451 (11)	0.0513 (12)	−0.0001 (10)	0.0160 (11)	−0.0001 (9)
Cl5	0.0886 (18)	0.0534 (13)	0.0864 (18)	0.0000 (12)	−0.0485 (14)	−0.0014 (12)
Cl6	0.0542 (12)	0.0547 (13)	0.0685 (14)	0.0109 (10)	−0.0023 (10)	0.0252 (11)
C53	0.051 (5)	0.050 (5)	0.038 (4)	0.011 (4)	0.005 (3)	0.006 (4)

### Geometric parameters (Å, °)

**Table:**

Ru1A—C45A	1.902 (6)	Ru1B—C44B	1.931 (6)
Ru1A—C46A	1.917 (6)	Ru1B—As1B	2.4267 (7)
Ru1A—C44A	1.930 (6)	Ru1B—Ru2B	2.8766 (7)
Ru1A—As1A	2.4238 (7)	Ru1B—Ru3B	2.8956 (6)
Ru1A—Ru3A	2.8688 (6)	Ru2B—C48B	1.881 (6)
Ru1A—Ru2A	2.8807 (6)	Ru2B—C49B	1.932 (6)
Ru2A—C48A	1.908 (6)	Ru2B—C47B	1.939 (6)
Ru2A—C49A	1.913 (6)	Ru2B—As2B	2.4201 (7)
Ru2A—C47A	1.937 (6)	Ru2B—Ru3B	2.8259 (6)
Ru2A—As2A	2.4313 (7)	Ru3B—C51B	1.884 (6)
Ru2A—Ru3A	2.8380 (6)	Ru3B—C50B	1.936 (6)
Ru3A—C51A	1.896 (6)	Ru3B—C52B	1.936 (6)
Ru3A—C52A	1.918 (6)	Ru3B—P1B	2.3262 (15)
Ru3A—C50A	1.946 (6)	As1B—C7B	1.936 (6)
Ru3A—P1A	2.3412 (15)	As1B—C1B	1.948 (6)
As1A—C7A	1.948 (5)	As1B—C13B	1.974 (5)
As1A—C13A	1.957 (5)	As2B—C14B	1.945 (5)
As1A—C1A	1.960 (5)	As2B—C20B	1.945 (6)
As2A—C14A	1.943 (6)	As2B—C13B	1.950 (5)
As2A—C20A	1.944 (5)	Cl1B—C29B	1.744 (6)
As2A—C13A	1.970 (5)	Cl2B—C35B	1.743 (6)
Cl1A—C29A	1.738 (6)	Cl3B—C41B	1.738 (6)
Cl2A—C35A	1.752 (6)	P1B—C26B	1.814 (6)
Cl3A—C41A	1.740 (6)	P1B—C32B	1.816 (6)
P1A—C26A	1.836 (5)	P1B—C38B	1.832 (6)
P1A—C32A	1.843 (5)	O1B—C44B	1.151 (7)
P1A—C38A	1.848 (6)	O2B—C45B	1.164 (8)
O1A—C44A	1.143 (7)	O3B—C46B	1.162 (7)
O2A—C45A	1.133 (7)	O4B—C47B	1.132 (7)
O3A—C46A	1.151 (7)	O5B—C48B	1.151 (7)
O4A—C47A	1.142 (7)	O6B—C49B	1.138 (7)
O5A—C48A	1.141 (7)	O7B—C50B	1.141 (7)
O6A—C49A	1.159 (7)	O8B—C51B	1.148 (7)
O7A—C50A	1.140 (7)	O9B—C52B	1.144 (7)
O8A—C51A	1.125 (6)	C1B—C6B	1.388 (8)
O9A—C52A	1.159 (7)	C1B—C2B	1.391 (8)
C1A—C2A	1.383 (8)	C2B—C3B	1.391 (9)
C1A—C6A	1.383 (8)	C2B—H2BA	0.9300
C2A—C3A	1.393 (8)	C3B—C4B	1.373 (9)
C2A—H2AA	0.9300	C3B—H3BA	0.9300
C3A—C4A	1.350 (9)	C4B—C5B	1.384 (9)
C3A—H3AA	0.9300	C4B—H4BA	0.9300
C4A—C5A	1.385 (9)	C5B—C6B	1.383 (8)
C4A—H4AA	0.9300	C5B—H5BA	0.9300
C5A—C6A	1.391 (8)	C6B—H6BA	0.9300
C5A—H5AA	0.9300	C7B—C8B	1.369 (9)
C6A—H6AA	0.9300	C7B—C12B	1.401 (8)
C7A—C12A	1.376 (8)	C8B—C9B	1.406 (9)
C7A—C8A	1.402 (8)	C8B—H8BA	0.9300
C8A—C9A	1.383 (9)	C9B—C10B	1.392 (11)
C8A—H8AA	0.9300	C9B—H9BA	0.9300
C9A—C10A	1.367 (10)	C10B—C11B	1.358 (10)
C9A—H9AA	0.9300	C10B—H10B	0.9300
C10A—C11A	1.396 (9)	C11B—C12B	1.389 (8)
C10A—H10A	0.9300	C11B—H11B	0.9300
C11A—C12A	1.397 (8)	C12B—H12B	0.9300
C11A—H11A	0.9300	C13B—H13C	0.9700
C12A—H12A	0.9300	C13B—H13D	0.9700
C13A—H13A	0.9700	C14B—C15B	1.382 (8)
C13A—H13B	0.9700	C14B—C19B	1.384 (8)
C14A—C15A	1.384 (8)	C15B—C16B	1.389 (9)
C14A—C19A	1.391 (8)	C15B—H15B	0.9300
C15A—C16A	1.380 (8)	C16B—C17B	1.337 (9)
C15A—H15A	0.9300	C16B—H16B	0.9300
C16A—C17A	1.386 (9)	C17B—C18B	1.403 (9)
C16A—H16A	0.9300	C17B—H17B	0.9300
C17A—C18A	1.372 (9)	C18B—C19B	1.394 (8)
C17A—H17A	0.9300	C18B—H18B	0.9300
C18A—C19A	1.390 (8)	C19B—H19B	0.9300
C18A—H18A	0.9300	C20B—C21B	1.390 (8)
C19A—H19A	0.9300	C20B—C25B	1.410 (8)
C20A—C25A	1.362 (8)	C21B—C22B	1.413 (8)
C20A—C21A	1.425 (8)	C21B—H21B	0.9300
C21A—C22A	1.374 (8)	C22B—C23B	1.383 (9)
C21A—H21A	0.9300	C22B—H22B	0.9300
C22A—C23A	1.361 (10)	C23B—C24B	1.373 (9)
C22A—H22A	0.9300	C23B—H23B	0.9300
C23A—C24A	1.378 (10)	C24B—C25B	1.375 (8)
C23A—H23A	0.9300	C24B—H24B	0.9300
C24A—C25A	1.414 (9)	C25B—H25B	0.9300
C24A—H24A	0.9300	C26B—C27B	1.367 (8)
C25A—H25A	0.9300	C26B—C31B	1.392 (8)
C26A—C31A	1.375 (8)	C27B—C28B	1.390 (8)
C26A—C27A	1.401 (8)	C27B—H27B	0.9300
C27A—C28A	1.370 (8)	C28B—C29B	1.366 (9)
C27A—H27A	0.9300	C28B—H28B	0.9300
C28A—C29A	1.391 (8)	C29B—C30B	1.362 (9)
C28A—H28A	0.9300	C30B—C31B	1.383 (8)
C29A—C30A	1.360 (8)	C30B—H30B	0.9300
C30A—C31A	1.390 (8)	C31B—H31B	0.9300
C30A—H30A	0.9300	C32B—C33B	1.388 (8)
C31A—H31A	0.9300	C32B—C37B	1.409 (8)
C32A—C33A	1.376 (7)	C33B—C34B	1.395 (9)
C32A—C37A	1.403 (7)	C33B—H33B	0.9300
C33A—C34A	1.386 (9)	C34B—C35B	1.358 (10)
C33A—H33A	0.9300	C34B—H34B	0.9300
C34A—C35A	1.383 (9)	C35B—C36B	1.389 (9)
C34A—H34A	0.9300	C36B—C37B	1.380 (8)
C35A—C36A	1.379 (8)	C36B—H36B	0.9300
C36A—C37A	1.371 (7)	C37B—H37B	0.9300
C36A—H36A	0.9300	C38B—C39B	1.370 (8)
C37A—H37A	0.9300	C38B—C43B	1.422 (8)
C38A—C39A	1.396 (7)	C39B—C40B	1.375 (8)
C38A—C43A	1.398 (8)	C39B—H39B	0.9300
C39A—C40A	1.386 (8)	C40B—C41B	1.355 (8)
C39A—H39A	0.9300	C40B—H40B	0.9300
C40A—C41A	1.386 (8)	C41B—C42B	1.382 (8)
C40A—H40A	0.9300	C42B—C43B	1.369 (8)
C41A—C42A	1.388 (8)	C42B—H42B	0.9300
C42A—C43A	1.393 (8)	C43B—H43B	0.9300
C42A—H42A	0.9300	Cl4—C53	1.723 (7)
C43A—H43A	0.9300	Cl5—C53	1.761 (8)
Ru1B—C45B	1.875 (6)	Cl6—C53	1.763 (7)
Ru1B—C46B	1.919 (6)	C53—H53A	0.9800
			
C45A—Ru1A—C46A	96.6 (3)	C45B—Ru1B—As1B	100.63 (19)
C45A—Ru1A—C44A	88.8 (2)	C46B—Ru1B—As1B	95.86 (17)
C46A—Ru1A—C44A	174.4 (2)	C44B—Ru1B—As1B	85.03 (17)
C45A—Ru1A—As1A	104.81 (19)	C45B—Ru1B—Ru2B	167.10 (19)
C46A—Ru1A—As1A	86.42 (19)	C46B—Ru1B—Ru2B	90.12 (17)
C44A—Ru1A—As1A	93.66 (16)	C44B—Ru1B—Ru2B	86.70 (19)
C45A—Ru1A—Ru3A	102.91 (19)	As1B—Ru1B—Ru2B	92.27 (2)
C46A—Ru1A—Ru3A	89.15 (18)	C45B—Ru1B—Ru3B	108.49 (19)
C44A—Ru1A—Ru3A	88.17 (16)	C46B—Ru1B—Ru3B	82.11 (17)
As1A—Ru1A—Ru3A	152.25 (2)	C44B—Ru1B—Ru3B	95.57 (17)
C45A—Ru1A—Ru2A	162.03 (19)	As1B—Ru1B—Ru3B	150.71 (3)
C46A—Ru1A—Ru2A	82.80 (19)	Ru2B—Ru1B—Ru3B	58.622 (15)
C44A—Ru1A—Ru2A	91.58 (16)	C48B—Ru2B—C49B	89.8 (3)
As1A—Ru1A—Ru2A	93.10 (2)	C48B—Ru2B—C47B	93.4 (3)
Ru3A—Ru1A—Ru2A	59.156 (15)	C49B—Ru2B—C47B	174.8 (2)
C48A—Ru2A—C49A	90.7 (3)	C48B—Ru2B—As2B	102.75 (18)
C48A—Ru2A—C47A	89.9 (3)	C49B—Ru2B—As2B	90.40 (17)
C49A—Ru2A—C47A	179.1 (3)	C47B—Ru2B—As2B	92.86 (17)
C48A—Ru2A—As2A	104.65 (17)	C48B—Ru2B—Ru3B	100.12 (19)
C49A—Ru2A—As2A	93.89 (17)	C49B—Ru2B—Ru3B	84.05 (17)
C47A—Ru2A—As2A	86.69 (18)	C47B—Ru2B—Ru3B	91.38 (17)
C48A—Ru2A—Ru3A	100.12 (17)	As2B—Ru2B—Ru3B	156.44 (3)
C49A—Ru2A—Ru3A	91.88 (17)	C48B—Ru2B—Ru1B	161.05 (19)
C47A—Ru2A—Ru3A	87.29 (18)	C49B—Ru2B—Ru1B	89.97 (17)
As2A—Ru2A—Ru3A	154.47 (3)	C47B—Ru2B—Ru1B	85.67 (18)
C48A—Ru2A—Ru1A	159.44 (17)	As2B—Ru2B—Ru1B	96.20 (2)
C49A—Ru2A—Ru1A	84.48 (17)	Ru3B—Ru2B—Ru1B	61.024 (16)
C47A—Ru2A—Ru1A	94.75 (19)	C51B—Ru3B—C50B	95.0 (2)
As2A—Ru2A—Ru1A	95.62 (2)	C51B—Ru3B—C52B	97.8 (2)
Ru3A—Ru2A—Ru1A	60.215 (16)	C50B—Ru3B—C52B	167.0 (2)
C51A—Ru3A—C52A	89.9 (2)	C51B—Ru3B—P1B	95.91 (18)
C51A—Ru3A—C50A	92.2 (2)	C50B—Ru3B—P1B	88.90 (17)
C52A—Ru3A—C50A	177.0 (2)	C52B—Ru3B—P1B	87.99 (17)
C51A—Ru3A—P1A	97.00 (17)	C51B—Ru3B—Ru2B	85.40 (18)
C52A—Ru3A—P1A	89.73 (17)	C50B—Ru3B—Ru2B	94.01 (17)
C50A—Ru3A—P1A	92.19 (17)	C52B—Ru3B—Ru2B	88.84 (17)
C51A—Ru3A—Ru2A	90.83 (17)	P1B—Ru3B—Ru2B	176.70 (4)
C52A—Ru3A—Ru2A	88.99 (17)	C51B—Ru3B—Ru1B	143.48 (18)
C50A—Ru3A—Ru2A	88.79 (17)	C50B—Ru3B—Ru1B	76.58 (17)
P1A—Ru3A—Ru2A	172.07 (4)	C52B—Ru3B—Ru1B	93.99 (16)
C51A—Ru3A—Ru1A	151.36 (17)	P1B—Ru3B—Ru1B	119.00 (4)
C52A—Ru3A—Ru1A	87.52 (17)	Ru2B—Ru3B—Ru1B	60.354 (16)
C50A—Ru3A—Ru1A	89.60 (17)	C7B—As1B—C1B	99.3 (2)
P1A—Ru3A—Ru1A	111.50 (4)	C7B—As1B—C13B	106.5 (2)
Ru2A—Ru3A—Ru1A	60.630 (15)	C1B—As1B—C13B	97.2 (2)
C7A—As1A—C13A	107.0 (2)	C7B—As1B—Ru1B	119.95 (17)
C7A—As1A—C1A	99.9 (2)	C1B—As1B—Ru1B	116.63 (18)
C13A—As1A—C1A	96.6 (2)	C13B—As1B—Ru1B	114.04 (16)
C7A—As1A—Ru1A	117.73 (16)	C14B—As2B—C20B	99.7 (2)
C13A—As1A—Ru1A	113.97 (16)	C14B—As2B—C13B	102.0 (2)
C1A—As1A—Ru1A	118.72 (18)	C20B—As2B—C13B	103.7 (2)
C14A—As2A—C20A	97.8 (2)	C14B—As2B—Ru2B	113.83 (17)
C14A—As2A—C13A	104.9 (2)	C20B—As2B—Ru2B	121.07 (16)
C20A—As2A—C13A	101.6 (2)	C13B—As2B—Ru2B	114.02 (16)
C14A—As2A—Ru2A	121.84 (17)	C26B—P1B—C32B	104.6 (3)
C20A—As2A—Ru2A	114.21 (17)	C26B—P1B—C38B	104.7 (2)
C13A—As2A—Ru2A	113.60 (16)	C32B—P1B—C38B	103.0 (3)
C26A—P1A—C32A	102.0 (3)	C26B—P1B—Ru3B	116.21 (18)
C26A—P1A—C38A	104.1 (2)	C32B—P1B—Ru3B	114.07 (19)
C32A—P1A—C38A	100.3 (2)	C38B—P1B—Ru3B	112.95 (19)
C26A—P1A—Ru3A	107.82 (17)	C6B—C1B—C2B	119.6 (6)
C32A—P1A—Ru3A	121.96 (18)	C6B—C1B—As1B	120.6 (5)
C38A—P1A—Ru3A	118.36 (19)	C2B—C1B—As1B	119.7 (5)
C2A—C1A—C6A	120.4 (5)	C1B—C2B—C3B	119.8 (6)
C2A—C1A—As1A	119.1 (4)	C1B—C2B—H2BA	120.1
C6A—C1A—As1A	120.5 (4)	C3B—C2B—H2BA	120.1
C1A—C2A—C3A	119.4 (6)	C4B—C3B—C2B	120.2 (6)
C1A—C2A—H2AA	120.3	C4B—C3B—H3BA	119.9
C3A—C2A—H2AA	120.3	C2B—C3B—H3BA	119.9
C4A—C3A—C2A	120.4 (6)	C3B—C4B—C5B	120.2 (6)
C4A—C3A—H3AA	119.8	C3B—C4B—H4BA	119.9
C2A—C3A—H3AA	119.8	C5B—C4B—H4BA	119.9
C3A—C4A—C5A	120.8 (6)	C6B—C5B—C4B	120.1 (6)
C3A—C4A—H4AA	119.6	C6B—C5B—H5BA	119.9
C5A—C4A—H4AA	119.6	C4B—C5B—H5BA	119.9
C4A—C5A—C6A	119.7 (6)	C5B—C6B—C1B	120.1 (6)
C4A—C5A—H5AA	120.1	C5B—C6B—H6BA	120.0
C6A—C5A—H5AA	120.1	C1B—C6B—H6BA	120.0
C1A—C6A—C5A	119.3 (6)	C8B—C7B—C12B	119.0 (6)
C1A—C6A—H6AA	120.3	C8B—C7B—As1B	123.9 (5)
C5A—C6A—H6AA	120.3	C12B—C7B—As1B	116.8 (4)
C12A—C7A—C8A	119.9 (5)	C7B—C8B—C9B	121.6 (7)
C12A—C7A—As1A	116.6 (4)	C7B—C8B—H8BA	119.2
C8A—C7A—As1A	123.5 (4)	C9B—C8B—H8BA	119.2
C9A—C8A—C7A	118.8 (6)	C10B—C9B—C8B	117.9 (7)
C9A—C8A—H8AA	120.6	C10B—C9B—H9BA	121.0
C7A—C8A—H8AA	120.6	C8B—C9B—H9BA	121.0
C10A—C9A—C8A	121.7 (6)	C11B—C10B—C9B	121.2 (6)
C10A—C9A—H9AA	119.2	C11B—C10B—H10B	119.4
C8A—C9A—H9AA	119.2	C9B—C10B—H10B	119.4
C9A—C10A—C11A	119.9 (6)	C10B—C11B—C12B	120.5 (7)
C9A—C10A—H10A	120.1	C10B—C11B—H11B	119.7
C11A—C10A—H10A	120.1	C12B—C11B—H11B	119.7
C10A—C11A—C12A	119.0 (6)	C11B—C12B—C7B	119.8 (6)
C10A—C11A—H11A	120.5	C11B—C12B—H12B	120.1
C12A—C11A—H11A	120.5	C7B—C12B—H12B	120.1
C7A—C12A—C11A	120.7 (6)	As2B—C13B—As1B	112.4 (3)
C7A—C12A—H12A	119.6	As2B—C13B—H13C	109.1
C11A—C12A—H12A	119.6	As1B—C13B—H13C	109.1
As1A—C13A—As2A	112.7 (3)	As2B—C13B—H13D	109.1
As1A—C13A—H13A	109.1	As1B—C13B—H13D	109.1
As2A—C13A—H13A	109.1	H13C—C13B—H13D	107.9
As1A—C13A—H13B	109.1	C15B—C14B—C19B	118.9 (5)
As2A—C13A—H13B	109.1	C15B—C14B—As2B	122.2 (4)
H13A—C13A—H13B	107.8	C19B—C14B—As2B	118.8 (5)
C15A—C14A—C19A	120.5 (5)	C14B—C15B—C16B	120.4 (6)
C15A—C14A—As2A	117.2 (4)	C14B—C15B—H15B	119.8
C19A—C14A—As2A	121.8 (4)	C16B—C15B—H15B	119.8
C16A—C15A—C14A	119.3 (6)	C17B—C16B—C15B	120.5 (6)
C16A—C15A—H15A	120.4	C17B—C16B—H16B	119.8
C14A—C15A—H15A	120.4	C15B—C16B—H16B	119.8
C15A—C16A—C17A	121.1 (6)	C16B—C17B—C18B	120.9 (6)
C15A—C16A—H16A	119.4	C16B—C17B—H17B	119.6
C17A—C16A—H16A	119.4	C18B—C17B—H17B	119.6
C18A—C17A—C16A	119.1 (6)	C19B—C18B—C17B	118.5 (6)
C18A—C17A—H17A	120.5	C19B—C18B—H18B	120.7
C16A—C17A—H17A	120.5	C17B—C18B—H18B	120.7
C17A—C18A—C19A	121.1 (6)	C14B—C19B—C18B	120.6 (6)
C17A—C18A—H18A	119.4	C14B—C19B—H19B	119.7
C19A—C18A—H18A	119.4	C18B—C19B—H19B	119.7
C18A—C19A—C14A	119.0 (6)	C21B—C20B—C25B	119.9 (5)
C18A—C19A—H19A	120.5	C21B—C20B—As2B	117.9 (4)
C14A—C19A—H19A	120.5	C25B—C20B—As2B	121.6 (4)
C25A—C20A—C21A	119.8 (5)	C20B—C21B—C22B	119.1 (6)
C25A—C20A—As2A	123.7 (4)	C20B—C21B—H21B	120.4
C21A—C20A—As2A	116.4 (4)	C22B—C21B—H21B	120.4
C22A—C21A—C20A	118.5 (6)	C23B—C22B—C21B	119.4 (6)
C22A—C21A—H21A	120.7	C23B—C22B—H22B	120.3
C20A—C21A—H21A	120.7	C21B—C22B—H22B	120.3
C23A—C22A—C21A	122.5 (6)	C24B—C23B—C22B	121.2 (6)
C23A—C22A—H22A	118.8	C24B—C23B—H23B	119.4
C21A—C22A—H22A	118.8	C22B—C23B—H23B	119.4
C22A—C23A—C24A	118.9 (6)	C23B—C24B—C25B	120.1 (6)
C22A—C23A—H23A	120.5	C23B—C24B—H24B	120.0
C24A—C23A—H23A	120.5	C25B—C24B—H24B	120.0
C23A—C24A—C25A	120.7 (6)	C24B—C25B—C20B	120.0 (6)
C23A—C24A—H24A	119.6	C24B—C25B—H25B	120.0
C25A—C24A—H24A	119.6	C20B—C25B—H25B	120.0
C20A—C25A—C24A	119.4 (6)	C27B—C26B—C31B	117.7 (5)
C20A—C25A—H25A	120.3	C27B—C26B—P1B	123.7 (4)
C24A—C25A—H25A	120.3	C31B—C26B—P1B	118.6 (4)
C31A—C26A—C27A	117.4 (5)	C26B—C27B—C28B	121.7 (6)
C31A—C26A—P1A	120.5 (4)	C26B—C27B—H27B	119.1
C27A—C26A—P1A	120.9 (4)	C28B—C27B—H27B	119.1
C28A—C27A—C26A	121.9 (6)	C29B—C28B—C27B	118.5 (6)
C28A—C27A—H27A	119.0	C29B—C28B—H28B	120.8
C26A—C27A—H27A	119.0	C27B—C28B—H28B	120.8
C27A—C28A—C29A	118.9 (5)	C30B—C29B—C28B	122.0 (6)
C27A—C28A—H28A	120.5	C30B—C29B—Cl1B	118.5 (5)
C29A—C28A—H28A	120.5	C28B—C29B—Cl1B	119.5 (5)
C30A—C29A—C28A	120.4 (5)	C29B—C30B—C31B	118.4 (6)
C30A—C29A—Cl1A	120.3 (5)	C29B—C30B—H30B	120.8
C28A—C29A—Cl1A	119.2 (4)	C31B—C30B—H30B	120.8
C29A—C30A—C31A	120.0 (6)	C30B—C31B—C26B	121.6 (6)
C29A—C30A—H30A	120.0	C30B—C31B—H31B	119.2
C31A—C30A—H30A	120.0	C26B—C31B—H31B	119.2
C26A—C31A—C30A	121.3 (5)	C33B—C32B—C37B	116.9 (6)
C26A—C31A—H31A	119.4	C33B—C32B—P1B	125.3 (5)
C30A—C31A—H31A	119.4	C37B—C32B—P1B	117.5 (4)
C33A—C32A—C37A	118.8 (5)	C32B—C33B—C34B	120.9 (6)
C33A—C32A—P1A	122.5 (4)	C32B—C33B—H33B	119.6
C37A—C32A—P1A	118.7 (4)	C34B—C33B—H33B	119.6
C32A—C33A—C34A	121.1 (5)	C35B—C34B—C33B	120.6 (6)
C32A—C33A—H33A	119.5	C35B—C34B—H34B	119.7
C34A—C33A—H33A	119.5	C33B—C34B—H34B	119.7
C35A—C34A—C33A	118.7 (5)	C34B—C35B—C36B	120.6 (6)
C35A—C34A—H34A	120.6	C34B—C35B—Cl2B	120.2 (6)
C33A—C34A—H34A	120.6	C36B—C35B—Cl2B	119.2 (6)
C36A—C35A—C34A	121.3 (5)	C37B—C36B—C35B	118.7 (6)
C36A—C35A—Cl2A	118.0 (4)	C37B—C36B—H36B	120.7
C34A—C35A—Cl2A	120.6 (5)	C35B—C36B—H36B	120.7
C37A—C36A—C35A	119.4 (5)	C36B—C37B—C32B	122.3 (6)
C37A—C36A—H36A	120.3	C36B—C37B—H37B	118.8
C35A—C36A—H36A	120.3	C32B—C37B—H37B	118.8
C36A—C37A—C32A	120.6 (5)	C39B—C38B—C43B	116.8 (5)
C36A—C37A—H37A	119.7	C39B—C38B—P1B	123.9 (5)
C32A—C37A—H37A	119.7	C43B—C38B—P1B	119.2 (4)
C39A—C38A—C43A	118.3 (5)	C38B—C39B—C40B	122.5 (6)
C39A—C38A—P1A	122.9 (4)	C38B—C39B—H39B	118.8
C43A—C38A—P1A	118.8 (4)	C40B—C39B—H39B	118.8
C40A—C39A—C38A	120.9 (5)	C41B—C40B—C39B	119.1 (6)
C40A—C39A—H39A	119.5	C41B—C40B—H40B	120.4
C38A—C39A—H39A	119.5	C39B—C40B—H40B	120.4
C41A—C40A—C39A	119.8 (5)	C40B—C41B—C42B	121.2 (6)
C41A—C40A—H40A	120.1	C40B—C41B—Cl3B	120.3 (5)
C39A—C40A—H40A	120.1	C42B—C41B—Cl3B	118.5 (5)
C40A—C41A—C42A	120.7 (6)	C43B—C42B—C41B	119.2 (6)
C40A—C41A—Cl3A	119.8 (4)	C43B—C42B—H42B	120.4
C42A—C41A—Cl3A	119.5 (5)	C41B—C42B—H42B	120.4
C41A—C42A—C43A	119.1 (6)	C42B—C43B—C38B	120.8 (5)
C41A—C42A—H42A	120.5	C42B—C43B—H43B	119.6
C43A—C42A—H42A	120.5	C38B—C43B—H43B	119.6
C42A—C43A—C38A	121.2 (5)	O1B—C44B—Ru1B	172.8 (5)
C42A—C43A—H43A	119.4	O2B—C45B—Ru1B	175.9 (6)
C38A—C43A—H43A	119.4	O3B—C46B—Ru1B	171.0 (5)
O1A—C44A—Ru1A	174.0 (5)	O4B—C47B—Ru2B	174.7 (5)
O2A—C45A—Ru1A	177.2 (6)	O5B—C48B—Ru2B	177.8 (6)
O3A—C46A—Ru1A	172.1 (5)	O6B—C49B—Ru2B	175.6 (5)
O4A—C47A—Ru2A	172.5 (6)	O7B—C50B—Ru3B	172.8 (5)
O5A—C48A—Ru2A	176.2 (6)	O8B—C51B—Ru3B	176.9 (5)
O6A—C49A—Ru2A	173.7 (5)	O9B—C52B—Ru3B	174.1 (5)
O7A—C50A—Ru3A	173.6 (5)	Cl4—C53—Cl5	111.0 (4)
O8A—C51A—Ru3A	178.0 (5)	Cl4—C53—Cl6	112.2 (4)
O9A—C52A—Ru3A	173.1 (5)	Cl5—C53—Cl6	109.1 (4)
C45B—Ru1B—C46B	88.4 (3)	Cl4—C53—H53A	108.1
C45B—Ru1B—C44B	94.5 (3)	Cl5—C53—H53A	108.1
C46B—Ru1B—C44B	176.7 (3)	Cl6—C53—H53A	108.1
			
C45A—Ru1A—Ru2A—C48A	−22.4 (8)	C45B—Ru1B—Ru2B—C48B	−9.0 (11)
C46A—Ru1A—Ru2A—C48A	−111.6 (6)	C46B—Ru1B—Ru2B—C48B	74.6 (7)
C44A—Ru1A—Ru2A—C48A	68.7 (6)	C44B—Ru1B—Ru2B—C48B	−104.7 (7)
As1A—Ru1A—Ru2A—C48A	162.4 (6)	As1B—Ru1B—Ru2B—C48B	170.4 (6)
Ru3A—Ru1A—Ru2A—C48A	−18.2 (6)	Ru3B—Ru1B—Ru2B—C48B	−6.1 (6)
C45A—Ru1A—Ru2A—C49A	−99.5 (6)	C45B—Ru1B—Ru2B—C49B	80.3 (9)
C46A—Ru1A—Ru2A—C49A	171.3 (3)	C46B—Ru1B—Ru2B—C49B	163.9 (2)
C44A—Ru1A—Ru2A—C49A	−8.4 (2)	C44B—Ru1B—Ru2B—C49B	−15.4 (2)
As1A—Ru1A—Ru2A—C49A	85.32 (18)	As1B—Ru1B—Ru2B—C49B	−100.25 (17)
Ru3A—Ru1A—Ru2A—C49A	−95.36 (18)	Ru3B—Ru1B—Ru2B—C49B	83.21 (17)
C45A—Ru1A—Ru2A—C47A	80.0 (6)	C45B—Ru1B—Ru2B—C47B	−96.9 (9)
C46A—Ru1A—Ru2A—C47A	−9.2 (3)	C46B—Ru1B—Ru2B—C47B	−13.3 (2)
C44A—Ru1A—Ru2A—C47A	171.1 (2)	C44B—Ru1B—Ru2B—C47B	167.4 (2)
As1A—Ru1A—Ru2A—C47A	−95.18 (18)	As1B—Ru1B—Ru2B—C47B	82.56 (17)
Ru3A—Ru1A—Ru2A—C47A	84.14 (18)	Ru3B—Ru1B—Ru2B—C47B	−93.99 (17)
C45A—Ru1A—Ru2A—As2A	167.1 (6)	C45B—Ru1B—Ru2B—As2B	170.7 (9)
C46A—Ru1A—Ru2A—As2A	77.94 (19)	C46B—Ru1B—Ru2B—As2B	−105.72 (18)
C44A—Ru1A—Ru2A—As2A	−101.81 (16)	C44B—Ru1B—Ru2B—As2B	75.03 (17)
As1A—Ru1A—Ru2A—As2A	−8.05 (2)	As1B—Ru1B—Ru2B—As2B	−9.85 (2)
Ru3A—Ru1A—Ru2A—As2A	171.27 (2)	Ru3B—Ru1B—Ru2B—As2B	173.60 (2)
C45A—Ru1A—Ru2A—Ru3A	−4.1 (6)	C45B—Ru1B—Ru2B—Ru3B	−2.9 (9)
C46A—Ru1A—Ru2A—Ru3A	−93.32 (19)	C46B—Ru1B—Ru2B—Ru3B	80.67 (17)
C44A—Ru1A—Ru2A—Ru3A	86.92 (16)	C44B—Ru1B—Ru2B—Ru3B	−98.57 (17)
As1A—Ru1A—Ru2A—Ru3A	−179.32 (2)	As1B—Ru1B—Ru2B—Ru3B	176.54 (2)
C48A—Ru2A—Ru3A—C51A	−8.8 (3)	C48B—Ru2B—Ru3B—C51B	−15.2 (3)
C49A—Ru2A—Ru3A—C51A	−99.9 (2)	C49B—Ru2B—Ru3B—C51B	73.5 (2)
C47A—Ru2A—Ru3A—C51A	80.5 (3)	C47B—Ru2B—Ru3B—C51B	−108.9 (2)
As2A—Ru2A—Ru3A—C51A	157.05 (17)	As2B—Ru2B—Ru3B—C51B	150.70 (18)
Ru1A—Ru2A—Ru3A—C51A	177.58 (17)	Ru1B—Ru2B—Ru3B—C51B	166.79 (17)
C48A—Ru2A—Ru3A—C52A	−98.7 (3)	C48B—Ru2B—Ru3B—C50B	−109.9 (3)
C49A—Ru2A—Ru3A—C52A	170.3 (2)	C49B—Ru2B—Ru3B—C50B	−21.2 (2)
C47A—Ru2A—Ru3A—C52A	−9.3 (3)	C47B—Ru2B—Ru3B—C50B	156.4 (2)
As2A—Ru2A—Ru3A—C52A	67.20 (19)	As2B—Ru2B—Ru3B—C50B	56.00 (18)
Ru1A—Ru2A—Ru3A—C52A	87.73 (18)	Ru1B—Ru2B—Ru3B—C50B	72.08 (17)
C48A—Ru2A—Ru3A—C50A	83.4 (3)	C48B—Ru2B—Ru3B—C52B	82.7 (3)
C49A—Ru2A—Ru3A—C50A	−7.7 (2)	C49B—Ru2B—Ru3B—C52B	171.5 (2)
C47A—Ru2A—Ru3A—C50A	172.7 (3)	C47B—Ru2B—Ru3B—C52B	−11.0 (2)
As2A—Ru2A—Ru3A—C50A	−110.74 (17)	As2B—Ru2B—Ru3B—C52B	−111.34 (18)
Ru1A—Ru2A—Ru3A—C50A	−90.22 (17)	Ru1B—Ru2B—Ru3B—C52B	−95.25 (17)
C48A—Ru2A—Ru3A—Ru1A	173.6 (2)	C48B—Ru2B—Ru3B—Ru1B	178.0 (2)
C49A—Ru2A—Ru3A—Ru1A	82.55 (18)	C49B—Ru2B—Ru3B—Ru1B	−93.27 (18)
C47A—Ru2A—Ru3A—Ru1A	−97.04 (19)	C47B—Ru2B—Ru3B—Ru1B	84.28 (18)
As2A—Ru2A—Ru3A—Ru1A	−20.53 (5)	As2B—Ru2B—Ru3B—Ru1B	−16.08 (6)
C45A—Ru1A—Ru3A—C51A	173.6 (4)	C45B—Ru1B—Ru3B—C51B	156.8 (4)
C46A—Ru1A—Ru3A—C51A	77.1 (4)	C46B—Ru1B—Ru3B—C51B	−117.5 (3)
C44A—Ru1A—Ru3A—C51A	−98.0 (4)	C44B—Ru1B—Ru3B—C51B	60.2 (3)
As1A—Ru1A—Ru3A—C51A	−3.6 (4)	As1B—Ru1B—Ru3B—C51B	−29.6 (3)
Ru2A—Ru1A—Ru3A—C51A	−5.1 (4)	Ru2B—Ru1B—Ru3B—C51B	−22.5 (3)
C45A—Ru1A—Ru3A—C52A	88.5 (3)	C45B—Ru1B—Ru3B—C50B	76.7 (3)
C46A—Ru1A—Ru3A—C52A	−8.1 (3)	C46B—Ru1B—Ru3B—C50B	162.4 (3)
C44A—Ru1A—Ru3A—C52A	176.8 (2)	C44B—Ru1B—Ru3B—C50B	−19.9 (3)
As1A—Ru1A—Ru3A—C52A	−88.78 (18)	As1B—Ru1B—Ru3B—C50B	−109.69 (18)
Ru2A—Ru1A—Ru3A—C52A	−90.23 (18)	Ru2B—Ru1B—Ru3B—C50B	−102.62 (18)
C45A—Ru1A—Ru3A—C50A	−92.5 (3)	C45B—Ru1B—Ru3B—C52B	−94.3 (3)
C46A—Ru1A—Ru3A—C50A	171.0 (3)	C46B—Ru1B—Ru3B—C52B	−8.6 (2)
C44A—Ru1A—Ru3A—C50A	−4.1 (2)	C44B—Ru1B—Ru3B—C52B	169.1 (3)
As1A—Ru1A—Ru3A—C50A	90.30 (17)	As1B—Ru1B—Ru3B—C52B	79.31 (18)
Ru2A—Ru1A—Ru3A—C50A	88.84 (17)	Ru2B—Ru1B—Ru3B—C52B	86.38 (17)
C45A—Ru1A—Ru3A—P1A	−0.3 (2)	C45B—Ru1B—Ru3B—P1B	−4.4 (2)
C46A—Ru1A—Ru3A—P1A	−96.8 (2)	C46B—Ru1B—Ru3B—P1B	81.30 (19)
C44A—Ru1A—Ru3A—P1A	88.10 (17)	C44B—Ru1B—Ru3B—P1B	−101.02 (19)
As1A—Ru1A—Ru3A—P1A	−177.51 (6)	As1B—Ru1B—Ru3B—P1B	169.22 (6)
Ru2A—Ru1A—Ru3A—P1A	−178.96 (4)	Ru2B—Ru1B—Ru3B—P1B	176.29 (5)
C45A—Ru1A—Ru3A—Ru2A	178.70 (19)	C45B—Ru1B—Ru3B—Ru2B	179.3 (2)
C46A—Ru1A—Ru3A—Ru2A	82.1 (2)	C46B—Ru1B—Ru3B—Ru2B	−94.99 (18)
C44A—Ru1A—Ru3A—Ru2A	−92.94 (16)	C44B—Ru1B—Ru3B—Ru2B	82.69 (18)
As1A—Ru1A—Ru3A—Ru2A	1.45 (5)	As1B—Ru1B—Ru3B—Ru2B	−7.08 (5)
C45A—Ru1A—As1A—C7A	81.1 (3)	C45B—Ru1B—As1B—C7B	79.3 (3)
C46A—Ru1A—As1A—C7A	177.0 (3)	C46B—Ru1B—As1B—C7B	−10.2 (3)
C44A—Ru1A—As1A—C7A	−8.6 (3)	C44B—Ru1B—As1B—C7B	173.0 (3)
Ru3A—Ru1A—As1A—C7A	−101.6 (2)	Ru2B—Ru1B—As1B—C7B	−100.5 (2)
Ru2A—Ru1A—As1A—C7A	−100.4 (2)	Ru3B—Ru1B—As1B—C7B	−94.5 (2)
C45A—Ru1A—As1A—C13A	−152.3 (3)	C45B—Ru1B—As1B—C1B	−40.5 (3)
C46A—Ru1A—As1A—C13A	−56.4 (3)	C46B—Ru1B—As1B—C1B	−130.0 (3)
C44A—Ru1A—As1A—C13A	118.0 (2)	C44B—Ru1B—As1B—C1B	53.1 (3)
Ru3A—Ru1A—As1A—C13A	24.9 (2)	Ru2B—Ru1B—As1B—C1B	139.59 (18)
Ru2A—Ru1A—As1A—C13A	26.17 (19)	Ru3B—Ru1B—As1B—C1B	145.64 (18)
C45A—Ru1A—As1A—C1A	−39.6 (3)	C45B—Ru1B—As1B—C13B	−152.7 (3)
C46A—Ru1A—As1A—C1A	56.3 (3)	C46B—Ru1B—As1B—C13B	117.8 (2)
C44A—Ru1A—As1A—C1A	−129.3 (2)	C44B—Ru1B—As1B—C13B	−59.1 (3)
Ru3A—Ru1A—As1A—C1A	137.67 (19)	Ru2B—Ru1B—As1B—C13B	27.43 (17)
Ru2A—Ru1A—As1A—C1A	138.92 (19)	Ru3B—Ru1B—As1B—C13B	33.47 (18)
C48A—Ru2A—As2A—C14A	−59.7 (3)	C48B—Ru2B—As2B—C14B	55.3 (3)
C49A—Ru2A—As2A—C14A	32.1 (3)	C49B—Ru2B—As2B—C14B	−34.6 (2)
C47A—Ru2A—As2A—C14A	−148.7 (3)	C47B—Ru2B—As2B—C14B	149.4 (3)
Ru3A—Ru2A—As2A—C14A	134.68 (19)	Ru3B—Ru2B—As2B—C14B	−110.52 (19)
Ru1A—Ru2A—As2A—C14A	116.88 (19)	Ru1B—Ru2B—As2B—C14B	−124.63 (18)
C48A—Ru2A—As2A—C20A	57.3 (3)	C48B—Ru2B—As2B—C20B	−63.5 (3)
C49A—Ru2A—As2A—C20A	149.1 (2)	C49B—Ru2B—As2B—C20B	−153.4 (3)
C47A—Ru2A—As2A—C20A	−31.6 (3)	C47B—Ru2B—As2B—C20B	30.6 (3)
Ru3A—Ru2A—As2A—C20A	−108.28 (19)	Ru3B—Ru2B—As2B—C20B	130.70 (19)
Ru1A—Ru2A—As2A—C20A	−126.09 (18)	Ru1B—Ru2B—As2B—C20B	116.59 (18)
C48A—Ru2A—As2A—C13A	173.3 (3)	C48B—Ru2B—As2B—C13B	171.7 (3)
C49A—Ru2A—As2A—C13A	−95.0 (3)	C49B—Ru2B—As2B—C13B	81.8 (2)
C47A—Ru2A—As2A—C13A	84.3 (3)	C47B—Ru2B—As2B—C13B	−94.2 (3)
Ru3A—Ru2A—As2A—C13A	7.65 (19)	Ru3B—Ru2B—As2B—C13B	5.88 (19)
Ru1A—Ru2A—As2A—C13A	−10.16 (18)	Ru1B—Ru2B—As2B—C13B	−8.23 (18)
C51A—Ru3A—P1A—C26A	11.5 (3)	C51B—Ru3B—P1B—C26B	142.9 (3)
C52A—Ru3A—P1A—C26A	101.3 (3)	C50B—Ru3B—P1B—C26B	−122.2 (3)
C50A—Ru3A—P1A—C26A	−81.0 (3)	C52B—Ru3B—P1B—C26B	45.2 (3)
Ru1A—Ru3A—P1A—C26A	−171.44 (19)	Ru1B—Ru3B—P1B—C26B	−48.2 (2)
C51A—Ru3A—P1A—C32A	128.7 (3)	C51B—Ru3B—P1B—C32B	−95.3 (3)
C52A—Ru3A—P1A—C32A	−141.4 (3)	C50B—Ru3B—P1B—C32B	−0.4 (3)
C50A—Ru3A—P1A—C32A	36.2 (3)	C52B—Ru3B—P1B—C32B	167.0 (3)
Ru1A—Ru3A—P1A—C32A	−54.2 (2)	Ru1B—Ru3B—P1B—C32B	73.6 (2)
C51A—Ru3A—P1A—C38A	−106.2 (3)	C51B—Ru3B—P1B—C38B	21.9 (3)
C52A—Ru3A—P1A—C38A	−16.3 (3)	C50B—Ru3B—P1B—C38B	116.8 (3)
C50A—Ru3A—P1A—C38A	161.3 (3)	C52B—Ru3B—P1B—C38B	−75.7 (3)
Ru1A—Ru3A—P1A—C38A	70.9 (2)	Ru1B—Ru3B—P1B—C38B	−169.19 (19)
C7A—As1A—C1A—C2A	50.1 (5)	C7B—As1B—C1B—C6B	−129.5 (5)
C13A—As1A—C1A—C2A	−58.5 (5)	C13B—As1B—C1B—C6B	122.5 (5)
Ru1A—As1A—C1A—C2A	179.5 (4)	Ru1B—As1B—C1B—C6B	1.0 (5)
C7A—As1A—C1A—C6A	−132.6 (5)	C7B—As1B—C1B—C2B	52.3 (5)
C13A—As1A—C1A—C6A	118.8 (5)	C13B—As1B—C1B—C2B	−55.8 (5)
Ru1A—As1A—C1A—C6A	−3.2 (5)	Ru1B—As1B—C1B—C2B	−177.3 (4)
C6A—C1A—C2A—C3A	0.9 (9)	C6B—C1B—C2B—C3B	−0.5 (9)
As1A—C1A—C2A—C3A	178.2 (4)	As1B—C1B—C2B—C3B	177.8 (5)
C1A—C2A—C3A—C4A	0.1 (9)	C1B—C2B—C3B—C4B	1.3 (10)
C2A—C3A—C4A—C5A	−1.4 (10)	C2B—C3B—C4B—C5B	−0.8 (10)
C3A—C4A—C5A—C6A	1.7 (10)	C3B—C4B—C5B—C6B	−0.4 (10)
C2A—C1A—C6A—C5A	−0.6 (9)	C4B—C5B—C6B—C1B	1.2 (9)
As1A—C1A—C6A—C5A	−177.8 (4)	C2B—C1B—C6B—C5B	−0.8 (9)
C4A—C5A—C6A—C1A	−0.7 (9)	As1B—C1B—C6B—C5B	−179.0 (5)
C13A—As1A—C7A—C12A	−177.3 (4)	C1B—As1B—C7B—C8B	−86.9 (6)
C1A—As1A—C7A—C12A	82.6 (5)	C13B—As1B—C7B—C8B	13.5 (6)
Ru1A—As1A—C7A—C12A	−47.4 (5)	Ru1B—As1B—C7B—C8B	144.9 (5)
C13A—As1A—C7A—C8A	4.6 (5)	C1B—As1B—C7B—C12B	87.0 (5)
C1A—As1A—C7A—C8A	−95.5 (5)	C13B—As1B—C7B—C12B	−172.5 (4)
Ru1A—As1A—C7A—C8A	134.5 (4)	Ru1B—As1B—C7B—C12B	−41.2 (5)
C12A—C7A—C8A—C9A	−1.3 (9)	C12B—C7B—C8B—C9B	0.7 (10)
As1A—C7A—C8A—C9A	176.7 (5)	As1B—C7B—C8B—C9B	174.5 (5)
C7A—C8A—C9A—C10A	0.6 (11)	C7B—C8B—C9B—C10B	−1.3 (11)
C8A—C9A—C10A—C11A	0.2 (11)	C8B—C9B—C10B—C11B	1.3 (11)
C9A—C10A—C11A—C12A	−0.4 (10)	C9B—C10B—C11B—C12B	−0.5 (11)
C8A—C7A—C12A—C11A	1.2 (9)	C10B—C11B—C12B—C7B	−0.2 (10)
As1A—C7A—C12A—C11A	−177.0 (5)	C8B—C7B—C12B—C11B	0.1 (9)
C10A—C11A—C12A—C7A	−0.3 (9)	As1B—C7B—C12B—C11B	−174.1 (5)
C7A—As1A—C13A—As2A	93.7 (3)	C14B—As2B—C13B—As1B	151.6 (3)
C1A—As1A—C13A—As2A	−163.8 (3)	C20B—As2B—C13B—As1B	−105.2 (3)
Ru1A—As1A—C13A—As2A	−38.3 (3)	Ru2B—As2B—C13B—As1B	28.5 (3)
C14A—As2A—C13A—As1A	−105.4 (3)	C7B—As1B—C13B—As2B	96.1 (3)
C20A—As2A—C13A—As1A	153.2 (3)	C1B—As1B—C13B—As2B	−161.9 (3)
Ru2A—As2A—C13A—As1A	30.0 (3)	Ru1B—As1B—C13B—As2B	−38.5 (3)
C20A—As2A—C14A—C15A	−97.4 (5)	C20B—As2B—C14B—C15B	−74.7 (5)
C13A—As2A—C14A—C15A	158.3 (4)	C13B—As2B—C14B—C15B	31.6 (5)
Ru2A—As2A—C14A—C15A	27.5 (5)	Ru2B—As2B—C14B—C15B	154.9 (4)
C20A—As2A—C14A—C19A	74.3 (5)	C20B—As2B—C14B—C19B	102.2 (5)
C13A—As2A—C14A—C19A	−30.0 (5)	C13B—As2B—C14B—C19B	−151.4 (4)
Ru2A—As2A—C14A—C19A	−160.8 (4)	Ru2B—As2B—C14B—C19B	−28.2 (5)
C19A—C14A—C15A—C16A	−1.3 (9)	C19B—C14B—C15B—C16B	−0.9 (9)
As2A—C14A—C15A—C16A	170.5 (4)	As2B—C14B—C15B—C16B	176.0 (5)
C14A—C15A—C16A—C17A	0.5 (9)	C14B—C15B—C16B—C17B	3.4 (9)
C15A—C16A—C17A—C18A	0.8 (9)	C15B—C16B—C17B—C18B	−3.0 (10)
C16A—C17A—C18A—C19A	−1.4 (9)	C16B—C17B—C18B—C19B	0.1 (10)
C17A—C18A—C19A—C14A	0.6 (9)	C15B—C14B—C19B—C18B	−2.0 (9)
C15A—C14A—C19A—C18A	0.8 (9)	As2B—C14B—C19B—C18B	−179.0 (5)
As2A—C14A—C19A—C18A	−170.6 (4)	C17B—C18B—C19B—C14B	2.4 (9)
C14A—As2A—C20A—C25A	−83.7 (5)	C14B—As2B—C20B—C21B	−97.5 (4)
C13A—As2A—C20A—C25A	23.4 (5)	C13B—As2B—C20B—C21B	157.5 (4)
Ru2A—As2A—C20A—C25A	146.1 (5)	Ru2B—As2B—C20B—C21B	28.0 (5)
C14A—As2A—C20A—C21A	93.6 (4)	C14B—As2B—C20B—C25B	73.5 (5)
C13A—As2A—C20A—C21A	−159.3 (4)	C13B—As2B—C20B—C25B	−31.5 (5)
Ru2A—As2A—C20A—C21A	−36.6 (5)	Ru2B—As2B—C20B—C25B	−161.0 (4)
C25A—C20A—C21A—C22A	−1.8 (9)	C25B—C20B—C21B—C22B	0.1 (8)
As2A—C20A—C21A—C22A	−179.2 (5)	As2B—C20B—C21B—C22B	171.3 (4)
C20A—C21A—C22A—C23A	−0.1 (10)	C20B—C21B—C22B—C23B	−1.4 (9)
C21A—C22A—C23A—C24A	2.4 (11)	C21B—C22B—C23B—C24B	3.7 (10)
C22A—C23A—C24A—C25A	−2.8 (11)	C22B—C23B—C24B—C25B	−4.8 (9)
C21A—C20A—C25A—C24A	1.4 (9)	C23B—C24B—C25B—C20B	3.4 (9)
As2A—C20A—C25A—C24A	178.6 (5)	C21B—C20B—C25B—C24B	−1.1 (8)
C23A—C24A—C25A—C20A	0.9 (10)	As2B—C20B—C25B—C24B	−171.9 (4)
C32A—P1A—C26A—C31A	−41.0 (5)	C32B—P1B—C26B—C27B	110.3 (5)
C38A—P1A—C26A—C31A	−144.9 (5)	C38B—P1B—C26B—C27B	2.3 (6)
Ru3A—P1A—C26A—C31A	88.5 (4)	Ru3B—P1B—C26B—C27B	−123.0 (5)
C32A—P1A—C26A—C27A	151.6 (5)	C32B—P1B—C26B—C31B	−70.8 (5)
C38A—P1A—C26A—C27A	47.7 (5)	C38B—P1B—C26B—C31B	−178.8 (5)
Ru3A—P1A—C26A—C27A	−78.9 (5)	Ru3B—P1B—C26B—C31B	55.9 (5)
C31A—C26A—C27A—C28A	1.7 (9)	C31B—C26B—C27B—C28B	1.7 (9)
P1A—C26A—C27A—C28A	169.5 (5)	P1B—C26B—C27B—C28B	−179.4 (5)
C26A—C27A—C28A—C29A	−1.2 (9)	C26B—C27B—C28B—C29B	1.0 (10)
C27A—C28A—C29A—C30A	1.0 (9)	C27B—C28B—C29B—C30B	−3.1 (10)
C27A—C28A—C29A—Cl1A	−179.5 (5)	C27B—C28B—C29B—Cl1B	175.4 (5)
C28A—C29A—C30A—C31A	−1.3 (9)	C28B—C29B—C30B—C31B	2.3 (10)
Cl1A—C29A—C30A—C31A	179.2 (5)	Cl1B—C29B—C30B—C31B	−176.2 (5)
C27A—C26A—C31A—C30A	−2.0 (8)	C29B—C30B—C31B—C26B	0.6 (10)
P1A—C26A—C31A—C30A	−169.8 (4)	C27B—C26B—C31B—C30B	−2.5 (10)
C29A—C30A—C31A—C26A	1.8 (9)	P1B—C26B—C31B—C30B	178.5 (5)
C26A—P1A—C32A—C33A	126.7 (5)	C26B—P1B—C32B—C33B	24.8 (6)
C38A—P1A—C32A—C33A	−126.3 (5)	C38B—P1B—C32B—C33B	134.0 (6)
Ru3A—P1A—C32A—C33A	6.7 (6)	Ru3B—P1B—C32B—C33B	−103.2 (6)
C26A—P1A—C32A—C37A	−52.8 (5)	C26B—P1B—C32B—C37B	−162.7 (4)
C38A—P1A—C32A—C37A	54.1 (5)	C38B—P1B—C32B—C37B	−53.5 (5)
Ru3A—P1A—C32A—C37A	−172.9 (4)	Ru3B—P1B—C32B—C37B	69.3 (5)
C37A—C32A—C33A—C34A	3.1 (9)	C37B—C32B—C33B—C34B	0.9 (10)
P1A—C32A—C33A—C34A	−176.5 (5)	P1B—C32B—C33B—C34B	173.5 (6)
C32A—C33A—C34A—C35A	−2.6 (9)	C32B—C33B—C34B—C35B	−2.0 (12)
C33A—C34A—C35A—C36A	0.9 (10)	C33B—C34B—C35B—C36B	1.7 (12)
C33A—C34A—C35A—Cl2A	178.6 (5)	C33B—C34B—C35B—Cl2B	−177.6 (6)
C34A—C35A—C36A—C37A	0.2 (9)	C34B—C35B—C36B—C37B	−0.5 (10)
Cl2A—C35A—C36A—C37A	−177.5 (5)	Cl2B—C35B—C36B—C37B	178.9 (5)
C35A—C36A—C37A—C32A	0.3 (9)	C35B—C36B—C37B—C32B	−0.5 (9)
C33A—C32A—C37A—C36A	−1.9 (8)	C33B—C32B—C37B—C36B	0.3 (9)
P1A—C32A—C37A—C36A	177.7 (4)	P1B—C32B—C37B—C36B	−172.8 (5)
C26A—P1A—C38A—C39A	24.5 (5)	C26B—P1B—C38B—C39B	89.1 (6)
C32A—P1A—C38A—C39A	−80.8 (5)	C32B—P1B—C38B—C39B	−20.0 (6)
Ru3A—P1A—C38A—C39A	144.1 (4)	Ru3B—P1B—C38B—C39B	−143.6 (5)
C26A—P1A—C38A—C43A	−157.9 (4)	C26B—P1B—C38B—C43B	−95.6 (5)
C32A—P1A—C38A—C43A	96.9 (5)	C32B—P1B—C38B—C43B	155.3 (5)
Ru3A—P1A—C38A—C43A	−38.2 (5)	Ru3B—P1B—C38B—C43B	31.8 (5)
C43A—C38A—C39A—C40A	0.5 (8)	C43B—C38B—C39B—C40B	−5.8 (10)
P1A—C38A—C39A—C40A	178.1 (4)	P1B—C38B—C39B—C40B	169.7 (5)
C38A—C39A—C40A—C41A	1.3 (9)	C38B—C39B—C40B—C41B	1.6 (11)
C39A—C40A—C41A—C42A	−2.3 (9)	C39B—C40B—C41B—C42B	4.5 (10)
C39A—C40A—C41A—Cl3A	175.5 (4)	C39B—C40B—C41B—Cl3B	−176.5 (5)
C40A—C41A—C42A—C43A	1.5 (9)	C40B—C41B—C42B—C43B	−6.0 (10)
Cl3A—C41A—C42A—C43A	−176.4 (5)	Cl3B—C41B—C42B—C43B	174.9 (5)
C41A—C42A—C43A—C38A	0.4 (9)	C41B—C42B—C43B—C38B	1.6 (9)
C39A—C38A—C43A—C42A	−1.3 (8)	C39B—C38B—C43B—C42B	4.1 (9)
P1A—C38A—C43A—C42A	−179.1 (5)	P1B—C38B—C43B—C42B	−171.6 (5)

### Hydrogen-bond geometry (Å, °)

**Table:**

Cg1 Cg2 and Cg3 are the centroids of the C14A–C19A, C20B–C25B and C1A–C6A benzene rings, respectively.

**Table:**

*D*—H···*A*	*D*—H	H···*A*	*D*···*A*	*D*—H···*A*
C5B—H5BA···O4B^i^	0.93	2.53	3.293 (8)	139
C23B—H23B···Cl1B^ii^	0.93	2.81	3.566 (7)	139
C40B—H40B···O3A^ii^	0.93	2.49	3.047 (8)	119
C4A—H4AA···Cg1^iii^	0.93	2.86	3.560 (7)	133
C4B—H4BA···Cg2^iv^	0.93	2.68	3.314 (7)	126
C16A—H16A···Cg3^v^	0.93	2.85	3.629 (7)	142
C16B—H16B···Cg2^vi^	0.93	2.94	3.591 (7)	128
C24A—H24A···Cg1^vii^	0.93	2.90	3.582 (7)	131

Symmetry codes: (i) −*x*+3/2, *y*, *z*+1/2; (ii) −*x*+2, −*y*+1, *z*−1/2; (iii) −*x*+2, −*y*+2, *z*−1/2; (iv) −*x*+1, −*y*+1, *z*+1/2; (v) *x*+3/2, −*y*, *z*; (vi) −*x*−1/2, *y*+1, *z*+1/2; (vii) −*x*+1/2, *y*+2, *z*+1/2.
